# The red cell distribution width to albumin ratio as a novel biomarker for predicting short-term mortality in severe pulmonary sepsis: a retrospective study with dual-cohort validation

**DOI:** 10.3389/fmed.2026.1805614

**Published:** 2026-04-07

**Authors:** Tianyi Zhang, Han Gao, Shasha Wang

**Affiliations:** Emergency Department, Binzhou Medical University Hospital, Binzhou, China

**Keywords:** biomarker, mortality, prognosis, pulmonary sepsis, red cell distribution width to albumin ratio

## Abstract

**Background:**

The Red Cell Distribution Width to Albumin Ratio (RAR) is a biomarker that reflects a patient’s nutritional status, inflammatory response, and oxidative stress, showing significant potential in critical care medicine. To investigate its prognostic value, we conducted a retrospective study using a dual-cohort design to assess the association between RAR and short-term (28-day) mortality in patients with pulmonary sepsis.

**Materials and methods:**

We retrospectively identified patients with sepsis secondary to pulmonary infections from the Binzhou Medical University Hospital medical records and the Medical Information Mart for Intensive Care (MIMIC-IV) database. To examine the association between RAR and short-term adverse outcomes in these patients, we employed several statistical methods, including Kaplan–Meier survival curves, multivariable Cox regression, and restricted cubic spline (RCS) analysis. Subsequently, we applied machine learning algorithms—namely the Boruta algorithm, LASSO-COX regression, and Random Forests—to identify the most predictive features. These features were then used to develop a final multivariable Cox regression model for risk prediction. The performance of this predictive model was evaluated using receiver operating characteristic (ROC) curve analysis.

**Result:**

The final analysis included 6,065 patients with pulmonary sepsis. The 28-day ICU and in-hospital mortality rates were 20.50 and 19.30%, respectively. In the fully adjusted multivariable model, a higher RAR was significantly associated with increased 28-day ICU and in-hospital mortality, whether treated as a continuous or categorical variable. For each unit increase in the continuous RAR score, the hazard ratios (HR) for 28-day ICU and in-hospital mortality were 1.52 (95% CI: 1.28–1.80) and 1.30 (95% CI: 1.09–1.55), respectively. Similarly, when compared to the low RAR group, the high RAR group had hazard ratios of 1.45 (95% CI: 1.23–1.70) and 1.29 (95% CI: 1.09–1.52) for the two outcomes. The restricted cubic spline (RCS) analysis revealed a positive dose–response relationship between RAR levels and short-term adverse outcomes. Furthermore, the risk prediction model incorporating RAR and eight other independent predictors demonstrated superior performance in identifying high-risk patients compared to traditional critical illness scoring systems, as shown by receiver operating characteristic (ROC) analysis. All findings were consistently validated in the external cohort.

**Conclusion:**

In conclusion, our study demonstrates a significant inverse association between the RAR and short-term survival in patients with severe pulmonary sepsis. The RAR-based scoring system we developed shows promise as a practical adjunct tool for clinical risk assessment. Prospective validation is warranted to confirm its utility in improving risk stratification for this patient population.

## Introduction

Sepsis is a life-threatening condition caused by a dysregulated host response to infection and is a leading cause of mortality in non-cardiac intensive care units (ICUs) (1, 2). This syndrome can be triggered by infections at various sites, with the lungs being one of the most common origins. Sepsis secondary to pulmonary infection, termed pulmonary sepsis, represents a severe complication of respiratory tract infections and poses a substantial burden on both patient health and public health systems (3–5). In recent years, the growing incidence of respiratory diseases, the widespread use of invasive respiratory procedures, and the increasing challenge of antimicrobial resistance have collectively contributed to a rising burden of pulmonary sepsis (6, 7). Therefore, elucidating the risk factors for sepsis following pulmonary infection and identifying reliable biomarkers for the early detection of high-risk patients are crucial for enhancing preventive measures and refining treatment strategies.

Research has established that a patient’s nutritional status, systemic inflammation, and oxidative stress levels significantly influence both the risk and clinical outcomes of pulmonary sepsis (8–10). Growing evidence confirms that malnutrition, inflammatory activation, and oxidative stress—as defined by specific biomarkers—are strongly associated with the incidence and mortality of pulmonary sepsis, underscoring the need for early risk assessment and intervention (11, 12). Serum albumin, the most abundant plasma carrier protein, is a key marker of nutritional and inflammatory status and fulfills vital physiological roles, including anti-inflammatory and antioxidant functions, coagulation regulation, and the maintenance of plasma osmotic pressure (13, 14). Consequently, low serum albumin levels are linked to impaired physiological function, the progression of various diseases, and increased all-cause mortality (15, 16). Separately, the red cell distribution width (RDW), a routine hematological parameter that measures the heterogeneity of red blood cell volume, serves as an integrative marker of multidimensional physiological dysfunction. An elevated RDW typically indicates impaired erythropoiesis or reduced red cell survival and is associated with pathological processes such as inflammation, oxidative stress, and malnutrition (17–19).

Critically, serum albumin and RDW collectively reflect the body’s inflammatory status, nutritional metabolism, and oxidative stress from distinct yet complementary perspectives. Recognizing their synergistic value in assessing physiological reserve and disease severity, the Red cell Distribution Width to Albumin Ratio (RAR) has recently been proposed as a novel integrative biomarker (20). RAR has demonstrated a superior ability to predict both malnutrition risk and adverse outcomes in various conditions, including heart failure, acute coronary syndrome, and stroke (21–23). However, evidence regarding the association between RAR and clinical outcomes in patients with pulmonary sepsis remains scarce. Therefore, this study was designed to investigate the relationship between RAR and prognosis in this specific patient population.

## Materials and methods

### Data source

This study employed a retrospective dual-cohort design. The primary analysis was conducted using data from the Medical Information Mart for Intensive Care (MIMIC-IV) database, a publicly available repository maintained by the Massachusetts Institute of Technology. MIMIC-IV contains comprehensive, de-identified clinical data from over 190,000 adult ICU patients admitted to a large U. S. academic medical center between 2008 and 2019. The use of this de-identified data for retrospective analysis was authorized under the database access protocol and was granted an exemption from ethical review (24). For external validation, a separate cohort was established, comprising critically ill patients with pulmonary sepsis admitted to the Emergency Intensive Care Unit (EICU) and Department of Critical Care Medicine (ICU) of Binzhou Medical University Hospital between March 2020 and March 2025.

### Study population

This study focused on patients with pulmonary sepsis during their first admission to the Intensive Care Unit (ICU). Cases were identified using International Classification of Diseases, Ninth and Tenth Revision (ICD-9/10) codes, which included specific diagnoses such as pneumococcal pneumonia, *Klebsiella pneumoniae* pneumonia, fungal pneumonia, viral pneumonia, Gram-negative bacterial pneumonia, aspiration pneumonia, and pneumonia of unspecified pathogen. The inclusion criteria were: (1) age ≥18 years; (2) first-time ICU admission; (3) ICU length of stay ≥24 h; (4) availability of complete Red cell Distribution Width (RDW) and Albumin (ALB) measurements; and (5) a Sequential Organ Failure Assessment (SOFA) score ≥2 at admission. Patients with a history of malignant tumors were excluded.

### Variable extraction

Data were extracted by executing Structured Query Language (SQL) commands using PostgreSQL (version 13.7.2) and Navicat Premium (version 16). The extracted variables were categorized into the following six groups: (1) demographic information; (2) comorbidities; (3) vital signs; (4) disease severity scores at admission; (5) laboratory parameters; and (6) treatment interventions. Detailed descriptions of all variables are provided in the baseline characteristics table.

### Definition of exposure and endpoints


$$\mathrm{RAR}=\log_{2}(1+\frac{\mathrm{Red}\;\text{blood cell distribution width}\;(\mathrm{RDW})}{\text{Albumin}\;(\mathrm{ALB})})$$

Patients were stratified into three groups (Q1–Q3) based on RAR tertiles. The primary endpoint of the study was 28-day ICU mortality, defined as death from any cause during the first ICU admission within 28 days of ICU entry. The secondary endpoint was 28-day in-hospital mortality, defined as death from any cause within 28 days of ICU admission, regardless of whether the patient was still in the ICU or had been transferred to a general ward.

### Subgroup analysis

We performed prespecified subgroup analyses to assess the consistency of the association across key patient characteristics. These included age (>65 vs. ≤65 years), sex, race, and comorbidities (hypertension, acute kidney injury [AKI], chronic kidney disease [CKD], heart failure [HF], diabetes mellitus [DM], hyperlipidemia [HLD], chronic bronchitis [CB], ischemic heart disease [IHD], and chronic obstructive pulmonary disease [COPD]). For each subgroup, a Cox proportional hazards model was used to calculate the hazard ratio (HR) and 95% confidence interval (CI), which were then visualized using forest plots.

### Association between RAR and outcomes

To investigate the association between RAR and primary and secondary endpoints, we constructed separate Cox proportional hazards regression models for continuous RAR and categorical RAR (quartiles). To account for potential confounding, we constructed three sequentially adjusted models: Model 1 was unadjusted; Model 2 was adjusted for demographic characteristics (age, sex, race, and weight) and baseline comorbidities; Model 3 was further adjusted for variables that showed significant differences between survivors and non-survivors, including laboratory values, vital signs, disease severity scores at admission, and treatment interventions (see Supplementary Tables S1–S3). To mitigate multicollinearity in Model 3, we calculated the variance inflation factor (VIF) for all included variables, excluding those with a VIF > 5 (Supplementary Table 4). The proportional hazards assumption was formally tested using Schoenfeld residuals for all covariates and for the global model. The proportional hazards assumption was formally tested using Schoenfeld residuals for all covariates and for the global model. The PH assumption tests yielded *p*-values > 0.05 for the global models and for the primary exposure variable RAR (Supplementary Figure S5), confirming that the proportional hazards assumption was satisfied (Supplementary Tables S5 and S6). To assess model stability, we calculated the events per variable (EPV) ratio for all multivariable models. An EPV ≥ 10 was considered indicative of adequate statistical power and minimal risk of overfitting. The large sample size (*N* = 6,065) and high event count (1,243 ICU deaths) in the primary cohort provided adequate statistical power to detect clinically meaningful associations. With approximately 30 events per covariate in the fully adjusted model, the study exceeded the recommended minimum of 10 events per variable, ensuring stable and reliable effect estimates. To flexibly model the potential non-linear relationship between RAR and short-term mortality, we performed restricted cubic spline (RCS) analysis with four knots placed at the 5th, 35th, 65th, and 95th percentiles of the RAR distribution. The median RAR value (2.71) was used as the reference. A formal test for non-linearity was conducted using a likelihood ratio test, comparing the model with the RCS terms to a model with RAR included as a linear term only. The RCS analysis was adjusted for the same covariates as in Model 3 to ensure consistency with the primary analyses. Finally, Kaplan–Meier survival curves were generated for supplementary visualization.

### Feature selection, risk prediction modeling, and validation

To develop a parsimonious risk prediction model for clinical application, patients in the internal MIMIC-IV cohort were randomly divided into training (70%) and testing (30%) sets. Based on the covariates included in Model 3, we employed an ensemble machine learning approach within the training set to identify the most robust predictors associated with the primary endpoint (28-day ICU mortality). First, we applied the Boruta algorithm, a random forest-based wrapper method that distinguishes truly important features from noise by comparing them with randomly permuted shadow features. This provided an initial ranking of all candidate variables. Second, to achieve model parsimony and mitigate overfitting, we applied LASSO (Least Absolute Shrinkage and Selection Operator) regression with 10-fold cross-validation, which shrinks the coefficients of less relevant variables to zero. Third, we used Random Survival Forests (RSF), an extension of the random forest algorithm that explicitly accounts for time-to-event data and censoring. RSF grows survival trees using log-rank splitting rules and calculates variable importance based on the increase in prediction error (Harrell’s C-index) when a variable is permuted in out-of-bag data. Variables that consistently ranked highly across these distinct methods were considered core predictors. This consensus-based approach ensured that our final variable selection was data-driven, reproducible, and not overly reliant on the bias of any single algorithm. By comparing the feature rankings and importance scores derived from these models, we systematically identified a set of core variables closely associated with the primary endpoint. Subsequently, multivariable Cox proportional hazards regression was applied to these core variables to identify those with independent prognostic significance. A prognostic prediction model was then constructed based on these variables (Table 1). The discriminative ability of the model was assessed using receiver operating characteristic (ROC) curves and the area under the curve (AUC). Finally, to validate the stability and generalizability of the constructed model, its predictive performance was independently tested on the internal testing set and the external validation cohort, and compared with traditional disease severity scores (SOFA, APS III, SAPS II, OASIS, SIRS, APACHE II).

**Table 1:** Summary descriptives table by groups of RAR group.

Variable	[ALL]	Q1	Q2	Q3	*p*-value
	*N* = 6,065	*N* = 2,022	*N* = 2,021	*N* = 2,022	
RAR	2.72 (0.35)	2.36 (0.13)	2.68 (0.09)	3.11 (0.24)	0.000
Age	67.0 (16.0)	65.4 (16.9)	68.3 (15.6)	67.2 (15.3)	<0.001
Gender:					<0.001
M	2,389 (39.4%)	720 (35.6%)	827 (40.9%)	842 (41.6%)	
F	3,676 (60.6%)	1,302 (64.4%)	1,194 (59.1%)	1,180 (58.4%)	
Race:					0.076
No-white	2,277 (37.5%)	798 (39.5%)	730 (36.1%)	749 (37.0%)	
White	3,788 (62.5%)	1,224 (60.5%)	1,291 (63.9%)	1,273 (63.0%)	
Weight (kg)	83.0 (25.8)	85.1 (24.6)	83.2 (26.6)	80.6 (25.9)	<0.001
HYP:					<0.001
No	4,000 (66.0%)	1,214 (60.0%)	1,349 (66.7%)	1,437 (71.1%)	
Yes	2,065 (34.0%)	808 (40.0%)	672 (33.3%)	585 (28.9%)	
AKI:					<0.001
No	2,366 (39.0%)	948 (46.9%)	750 (37.1%)	668 (33.0%)	
Yes	3,699 (61.0%)	1,074 (53.1%)	1,271 (62.9%)	1,354 (67.0%)	
CKD:					<0.001
No	4,593 (75.7%)	1,615 (79.9%)	1,465 (72.5%)	1,513 (74.8%)	
Yes	1,472 (24.3%)	407 (20.1%)	556 (27.5%)	509 (25.2%)	
DM:					<0.001
No	4,148 (68.4%)	1,445 (71.5%)	1,315 (65.1%)	1,388 (68.6%)	
Yes	1,917 (31.6%)	577 (28.5%)	706 (34.9%)	634 (31.4%)	
HLD:					<0.001
No	4,071 (67.1%)	1,286 (63.6%)	1,347 (66.7%)	1,438 (71.1%)	
Yes	1,994 (32.9%)	736 (36.4%)	674 (33.3%)	584 (28.9%)	
CB:					0.129
No	5,232 (86.3%)	1,750 (86.5%)	1,719 (85.1%)	1,763 (87.2%)	
Yes	833 (13.7%)	272 (13.5%)	302 (14.9%)	259 (12.8%)	
HF:					0.010
No	3,743 (61.7%)	1,255 (62.1%)	1,197 (59.2%)	1,291 (63.8%)	
Yes	2,322 (38.3%)	767 (37.9%)	824 (40.8%)	731 (36.2%)	
IHD:					0.020
No	3,940 (65.0%)	1,283 (63.5%)	1,295 (64.1%)	1,362 (67.4%)	
Yes	2,125 (35.0%)	739 (36.5%)	726 (35.9%)	660 (32.6%)	
COPD:					0.006
No	4,772 (78.7%)	1,620 (80.1%)	1,542 (76.3%)	1,610 (79.6%)	
Yes	1,293 (21.3%)	402 (19.9%)	479 (23.7%)	412 (20.4%)	
SOFA	7.31 (3.60)	6.50 (3.28)	7.29 (3.55)	8.13 (3.78)	<0.001
APSIII	58.3 (22.0)	51.5 (20.2)	58.2 (21.4)	65.2 (22.2)	<0.001
SIRS	2.90 (0.86)	2.81 (0.88)	2.89 (0.87)	3.00 (0.83)	<0.001
SAPSII	44.4 (14.4)	40.5 (13.6)	44.9 (14.0)	47.7 (14.5)	<0.001
OASIS	36.5 (8.47)	35.1 (8.15)	36.5 (8.49)	37.8 (8.55)	<0.001
APACHEII	21.8 (7.31)	19.9 (7.20)	22.2 (7.12)	23.4 (7.19)	<0.001
HR (beats/min)	93.9 (21.8)	89.9 (20.5)	94.2 (22.1)	97.4 (22.0)	<0.001
NBPS (mmHg)	120 (25.3)	125 (25.3)	121 (25.1)	115 (24.6)	<0.001
NBPD (mmHg)	70.1 (89.4)	71.9 (19.2)	68.3 (19.5)	70.0 (152)	<0.001
RR (insp/min)	21.4 (6.97)	20.8 (6.59)	21.4 (6.59)	22.0 (7.62)	<0.001
SpO_2_ (%)	96.1 (12.1)	96.1 (4.68)	96.3 (19.8)	95.9 (4.83)	0.215
TF (°F)	98.2 (4.10)	98.2 (4.53)	98.3 (3.13)	98.0 (4.47)	0.022
HCT (%)	32.1 (7.19)	35.2 (7.02)	31.9 (6.76)	29.3 (6.52)	<0.001
Hb (g/dL)	10.4 (2.38)	11.6 (2.34)	10.3 (2.20)	9.35 (2.06)	<0.001
PLT (K/μL)	204 (121)	204 (97.5)	203 (119)	204 (141)	0.980
RDW (%)	15.9 (2.65)	14.2 (1.41)	15.7 (1.92)	17.9 (2.90)	0.000
RBC (m/uL)	3.50 (0.84)	3.81 (0.80)	3.48 (0.81)	3.20 (0.79)	<0.001
WBC (K/μL)	13.9 (12.3)	13.3 (10.5)	13.8 (13.3)	14.7 (12.9)	0.001
ALB (g/dL)	2.90 (0.61)	3.45 (0.42)	2.89 (0.36)	2.35 (0.44)	0.000
AG (mEq/L)	15.4 (4.94)	15.6 (4.83)	15.4 (4.98)	15.1 (5.01)	0.012
Tca (mg/dL)	8.22 (0.96)	8.46 (0.88)	8.24 (0.94)	7.97 (0.98)	<0.001
Cl (mEq/L)	103 (7.73)	102 (7.14)	103 (7.82)	104 (8.14)	<0.001
Glu (mg/dL)	154 (85.2)	158 (81.4)	156 (84.3)	148 (89.5)	<0.001
K (mEq/L)	4.27 (0.82)	4.27 (0.82)	4.30 (0.80)	4.25 (0.85)	0.121
Na (mEq/L)	138 (6.46)	138 (5.87)	139 (6.65)	138 (6.81)	0.129
TCO_2_ (mEq/L)	24.8 (6.37)	25.1 (6.18)	25.1 (6.43)	24.2 (6.45)	<0.001
Lac (mmol/L)	2.44 (2.13)	2.25 (1.85)	2.39 (2.11)	2.69 (2.39)	<0.001
PCO_2_ (mmHg)	44.7 (14.0)	44.7 (14.1)	45.3 (14.3)	44.0 (13.6)	0.013
PH (pH units)	7.34 (0.11)	7.35 (0.11)	7.34 (0.11)	7.34 (0.11)	0.003
PO_2_ (mmHg)	107 (90.1)	116 (96.1)	106 (90.6)	98.9 (82.3)	<0.001
INR (ratio)	1.67 (1.12)	1.54 (1.06)	1.68 (1.11)	1.80 (1.16)	<0.001
PT (seconds)	18.1 (11.2)	16.7 (10.4)	18.2 (11.3)	19.5 (11.7)	<0.001
PTT (seconds)	39.9 (24.3)	38.9 (24.9)	38.8 (23.2)	42.0 (24.7)	<0.001
ALT (IU/L)	135 (552)	160 (706)	137 (507)	108 (399)	0.007
AST (IU/L)	221 (911)	238 (1,049)	227 (909)	200 (752)	0.346
TB (mg/dL)	1.92 (4.54)	1.35 (3.56)	1.78 (4.35)	2.64 (5.42)	<0.001
CRE (mg/dL)	1.78 (1.79)	1.63 (1.80)	1.87 (1.86)	1.84 (1.68)	<0.001
URE (mg/dL)	34.2 (26.3)	28.8 (23.4)	35.8 (27.4)	38.0 (27.0)	<0.001
LDH (U/L)	537 (1,082)	507 (913)	559 (1,240)	544 (1,069)	0.256
Mg (mg/dL)	2.00 (0.47)	2.02 (0.48)	2.00 (0.47)	1.99 (0.45)	0.141
Lymphocyte count (%)	10.7 (10.3)	11.1 (9.53)	10.9 (10.2)	9.96 (11.0)	0.001
CRRT:					<0.001
No	5,301 (87.4%)	1,842 (91.1%)	1,774 (87.8%)	1,685 (83.3%)	
Yes	764 (12.6%)	180 (8.90%)	247 (12.2%)	337 (16.7%)	
Ventilation:					0.001
No	426 (7.02%)	108 (5.34%)	148 (7.32%)	170 (8.41%)	
Yes	5,639 (93.0%)	1,914 (94.7%)	1,873 (92.7%)	1,852 (91.6%)	
Sa:					0.245
No	1,357 (22.4%)	429 (21.2%)	473 (23.4%)	455 (22.5%)	
Yes	4,708 (77.6%)	1,593 (78.8%)	1,548 (76.6%)	1,567 (77.5%)	
GC:					0.002
No	3,472 (57.2%)	1,217 (60.2%)	1,150 (56.9%)	1,105 (54.6%)	
Yes	2,593 (42.8%)	805 (39.8%)	871 (43.1%)	917 (45.4%)	
VP:					<0.001
No	1,866 (30.8%)	744 (36.8%)	641 (31.7%)	481 (23.8%)	
Yes	4,199 (69.2%)	1,278 (63.2%)	1,380 (68.3%)	1,541 (76.2%)	
ABX:					0.037
No	4 (0.07%)	4 (0.20%)	0 (0.00%)	0 (0.00%)	
Yes	6,061 (99.9%)	2,018 (99.8%)	2,021 (100%)	2,022 (100%)	
Hosp_Time	18.9 (18.3)	17.9 (16.5)	18.8 (18.0)	20.2 (20.0)	<0.001
Hosp_dead	1,170 (19.3%)	271 (13.4%)	381 (18.9%)	518 (25.6%)	<0.001
ICU_Time	8.12 (9.01)	8.53 (8.85)	8.03 (9.26)	7.81 (8.91)	0.031
ICU_dead	1,243 (20.5%)	287 (14.2%)	405 (20.0%)	551 (27.3%)	<0.001

AKI, Acute Kidney Injury; CKD, Chronic Kidney Disease; Hld, Hyperlipidemia; CB, Chronic Bronchitis; HF, Heart Failure; IHD, Ischemic Heart Disease; COPD, Chronic Obstructive Pulmonary Disease; BMI, Body mass index; SOFA, Sequential Organ Failure Assessment; APSIII, Acute Physiology and Chronic Health III Score; SIRS, Systemic Inflammatory Response Syndrome Score; SAPSII, Simplified Acute Physiology Score II; OASIS, Oxford Acute Severity of Illness Score; APACHII, Acute Physiology and Chronic Health Evaluation II; HR, Heart Rate; NBPS, Non-invasive Blood Pressure Systolic; RR, Respiratory Rate; NBPD, Non invasive diastolic blood pressure; SPO2, Oxygen saturation; HCT, Hematocrit; Hb, Hemoglobin; PLT, Platelet; RDW, Red Blood Cell Distribution Width; RBC, Red blood cell count; WBC, White Blood Cell; ALB, Albumin; AG, Anion Gap; Glu, Glucose; K, Blood potassium; Na, Blood sodium; Mg, Blood magnesium; TCO2, Total amount of carbon dioxide; PCO2, Partial pressure of carbon dioxide; LAC, Lactate; PH, acidity and alkalinity; PO2, Oxygen partial pressure; INR, International Normalized Ratio; PT, Prothrombin Time; APTT, Activated Partial Thromboplastin Time; ALT, Alanine Aminotransferase; AST, Aspartate Aminotransferase; TB, Total Bilirubin; CRE, Creatinine; UREA, Urea Nitrogen; Mg, magnesium; CRRT, Continuous Renal Replacement Therapy; Ventilation, Mechanical Ventilation; GC, Corticosteroids; ABX, Antibiotics; VP, Vasoactive drugs; Sa, Analgesic and sedative drugs.

### Statistical analysis

Continuous variables are presented as mean ± standard deviation (SD) or median with interquartile range (IQR), as appropriate. Group comparisons for continuous data were made using the Student’s t-test or analysis of variance (ANOVA) for normally distributed data, and the Mann–Whitney U or Kruskal-Wallis test for non-normally distributed data. Categorical variables are expressed as frequencies and percentages, and comparisons were made using the Pearson chi-square test or Fisher’s exact test. All statistical analyses were performed using R software (version 4.2.2). A two-sided *p*-value of less than 0.05 was considered statistically significant.

## Result

### Baseline characteristics of study participants

The 6,065 patients with pulmonary sepsis were stratified into three groups based on RAR tertiles for baseline characterization. As shown in Table 1, higher RAR levels (Q1–Q3: 2.36–3.11) revealed a clear gradient in clinical severity. Assessments of disease severity consistently demonstrated that patients in higher RAR tertiles were more critically ill, as reflected by increasing scores across all systems: the SOFA score rose from 6.50 ± 3.28 to 8.13 ± 3.78, the APACHE II score from 19.9 ± 7.20 to 23.4 ± 7.19, and similar trends were observed for APS III, SAPS II, and OASIS scores (all *p* < 0.001). Laboratory findings confirmed the expected changes in the components of RAR, with RDW increasing from 14.2% ± 1.41 to 17.9% ± 2.90% and serum albumin decreasing from 3.45 ± 0.42 g/dL to 2.35 ± 0.44 g/dL (both p < 0.001). Furthermore, elevated RAR was associated with progressive declines in hemoglobin (11.6–9.35 g/dL) and red blood cell count (3.81 to 3.20 × 10^12^/L), alongside worsening inflammation (elevated white blood cell count and lactate) and impaired coagulation (prolonged INR, PT, and PTT). Clinical outcomes were significantly worse in the high-RAR group. The Q3 group had a hospital mortality rate of 25.6%, more than double the 13.4% rate in the Q1 group (*p* < 0.001). A similar increasing trend was observed for ICU mortality (27.3% vs. 14.2%, p < 0.001). Additionally, patients with higher RAR required more frequent interventions, including continuous renal replacement therapy (16.7% vs. 8.90%) and vasopressor support (76.2% vs. 63.2%; all p < 0.001).

### Multivariable cox regression analysis of RAR and short-term mortality in pulmonary Sepsis

**Figure 1:**
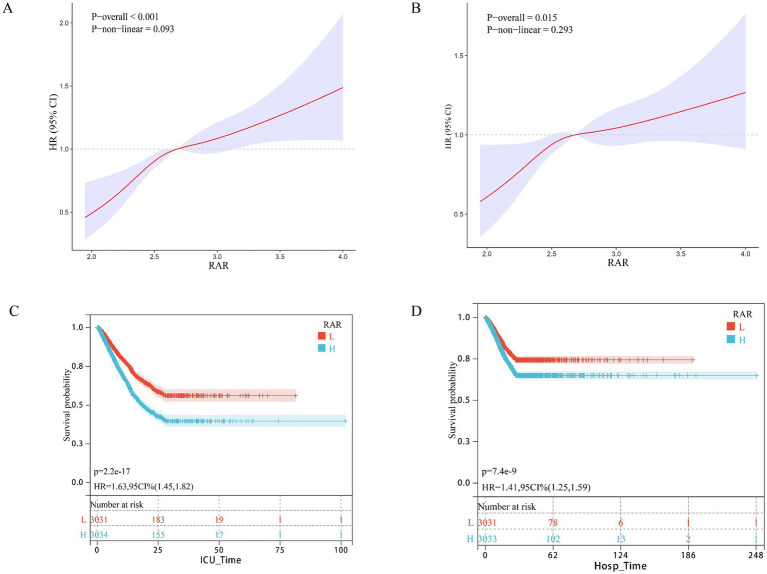
Association between RAR and 28-day survival in the internal cohort. **(A, B)** Restricted cubic spline (RCS) curves show the nonlinear relationship between continuous RAR score and the odds of **(A)** 28-day ICU mortality and **(B)** 28-day in-hospital mortality. **(C, D)** Kaplan–Meier survival curves illustrate the probability of survival from **(C)** ICU death and **(D)** in-hospital death 28 days, stratified by RAR score stratification (blue; low RAR score, high RAR score, red). *p*-value and hazard ratios (HR) with 95% confidence intervals are derived from Cox proportional hazards models.

As detailed in Table 2, a higher RAR was significantly and independently associated with increased 28-day ICU and in-hospital mortality in patients with pulmonary sepsis, both as a continuous variable and when categorized into tertiles. In the unadjusted model (Model 1), each unit increase in RAR was associated with a 141% higher risk of 28-day ICU mortality (HR 2.41, 95% CI 2.09–2.79). Analysis by RAR tertiles showed that compared to the Q1 group, the Q2 and Q3 groups had a 54% (HR 1.54, 95% CI 1.32–1.79) and 113% (HR 2.13, 95% CI 1.84–2.45) increased risk of ICU mortality, respectively (*p* < 0.001 for both). This association, though attenuated, remained statistically significant after sequential adjustment for confounders. In the fully adjusted model (Model 3), each unit increase in RAR was associated with a 52% increased risk of ICU mortality (HR 1.52, 95% CI 1.28–1.80). Similarly, the risk was 45% higher in the Q3 group (HR 1.45, 95% CI 1.23–1.70) and 26% higher in the Q2 group (HR 1.26, 95% CI 1.08–1.48) compared to Q1. RAR was also an independent predictor of 28-day in-hospital mortality. In the fully adjusted Model 3, each unit increase in RAR was associated with a 30% higher risk (HR 1.30, 95% CI 1.09–1.55), and the Q3 group had a 29% increased risk (HR 1.29, 95% CI 1.09–1.52) compared to Q1. Restricted cubic spline (RCS) analysis revealed a positive dose–response relationship between RAR and short-term mortality (Figures 1A,B). The likelihood ratio tests for non-linearity were not statistically significant for either 28-day ICU mortality (P for non-linearity = 0.234) or 28-day in-hospital mortality (P for non-linearity = 0.187), indicating that the relationship did not significantly deviate from linearity. The RCS curves visually confirm the consistent increase in mortality risk across the full spectrum of RAR values. Kaplan–Meier survival curves corroborated these findings, demonstrating significantly lower survival probabilities for patients in higher RAR tertiles (Figures 1C,D).

**Table 2:** The relationship between RAR score and 28 day ICU/hospital mortality rate.

Characteristic	Model 1			Model 2			Model 3		
	HR	95% CI	*p*-value	HR	95% CI	*p*-value	HR	95% CI	*p*-value
RAR	2.41	2.09, 2.79	<0.001	2.13	1.83, 2.46	<0.001	1.52	1.28, 1.80	<0.001
RAR group									
Q1	Ref	Ref		Ref	Ref		Ref	Ref	
Q2	1.54	1.32, 1.79	<0.001	1.41	1.21, 1.64	<0.001	1.26	1.08, 1.48	0.004
Q3	2.13	1.84, 2.45	<0.001	1.88	1.63, 2.17	<0.001	1.45	1.23, 1.70	<0.001
RAR	1.98	1.71, 2.30	<0.001	1.77	1.52, 2.06	<0.001	1.30	1.09, 1.55	0.004
RAR group									
Q1	Ref	Ref		Ref	Ref		Ref	Ref	
Q2	1.35	1.16, 1.58	<0.001	1.24	1.06, 1.45	0.007	1.12	0.96, 1.32	0.2
Q3	1.79	1.54, 2.07	<0.001	1.61	1.39, 1.87	<0.001	1.29	1.09, 1.52	0.003

CI, Confidence Interval; HR, Hazard Ratio, Ref, reference. Model 1: Unadjusted. Model 2: Adjusted for demographic characteristics and baseline comorbidities. This included: Age, Gender, Race, Weight, Acute Kidney Injury (AKI), Diabetes Mellitus (DM), and Hyperlipidemia (HLD). Model 3: Adjusted for all variables in Model 2, plus: SOFA, APSIII, SIRS, SAPSII, OASIS, APACHEII. Heart Rate (HR), Non-invasive Blood Pressure Systolic (NBPS), Respiratory Rate (RR), Oxygen Saturation (SPO2), Temperature (Fahrenheit). Platelet count (PLT), Red Blood Cell count (RBC), White Blood Cell count (WBC), Total Calcium (Tca), Anion Gap (AG), Glucose (Glu), Total Carbon Dioxide (TCO2), Lactate (Lac), pH, Partial Pressure of Oxygen (PO2), Prothrombin Time (PT), Activated Partial Thromboplastin Time (APTT), Alanine Aminotransferase (ALT), Total Bilirubin (TB), Urea Nitrogen (URE), Lactate Dehydrogenase (LDH), Magnesium (Mg), and Lymphocyte count. Treatment Interventions: Continuous Renal Replacement Therapy (CRRT), Mechanical Ventilation, Sedatives/Analgesics (Sa), Glucocorticoids (GC), and Vasopressors (VP).

### Subgroup analysis

**Figure 2:**
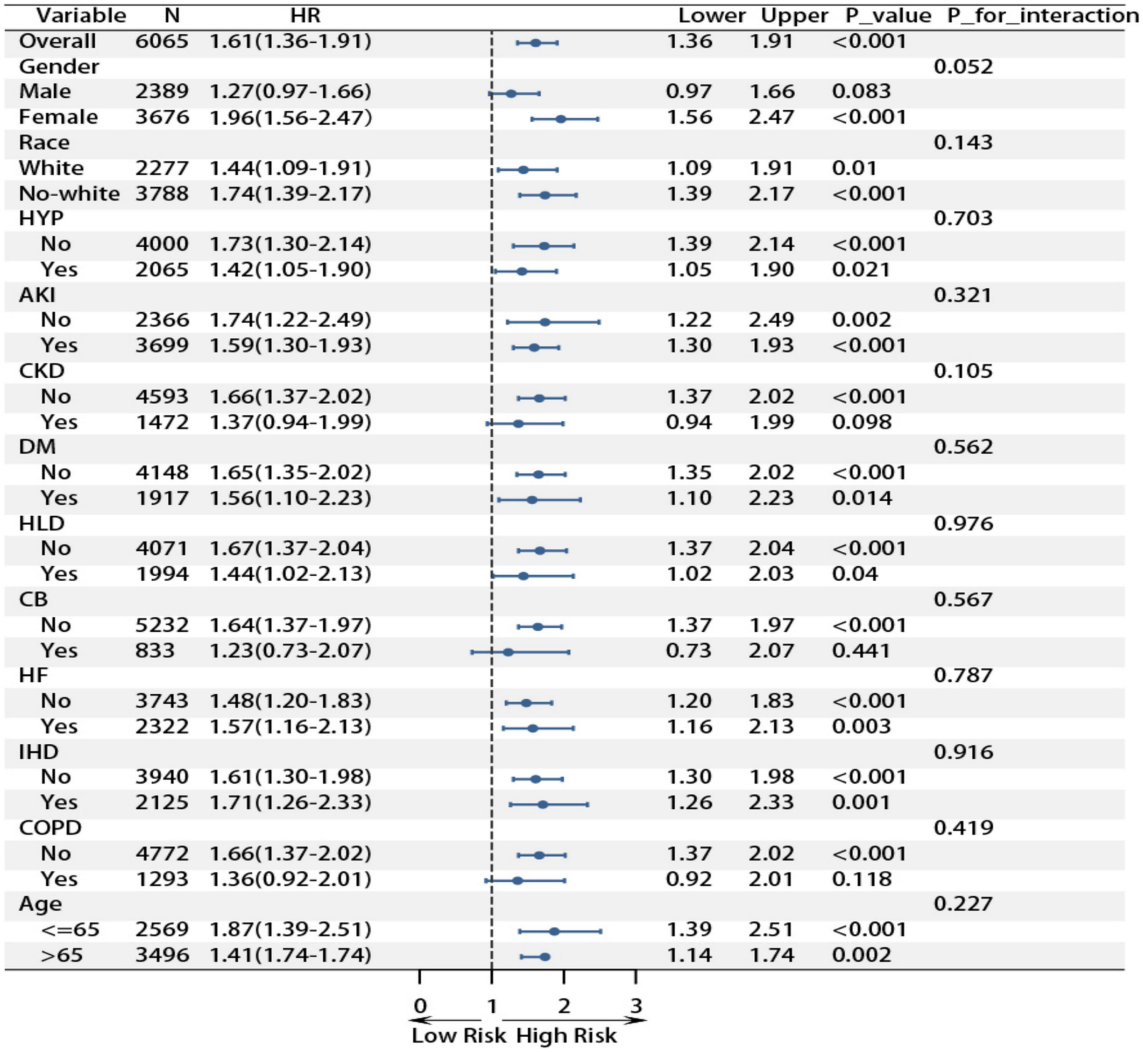
Subgroup analysis of RAR and 28 day ICU mortality rate.

**Figure 3:**
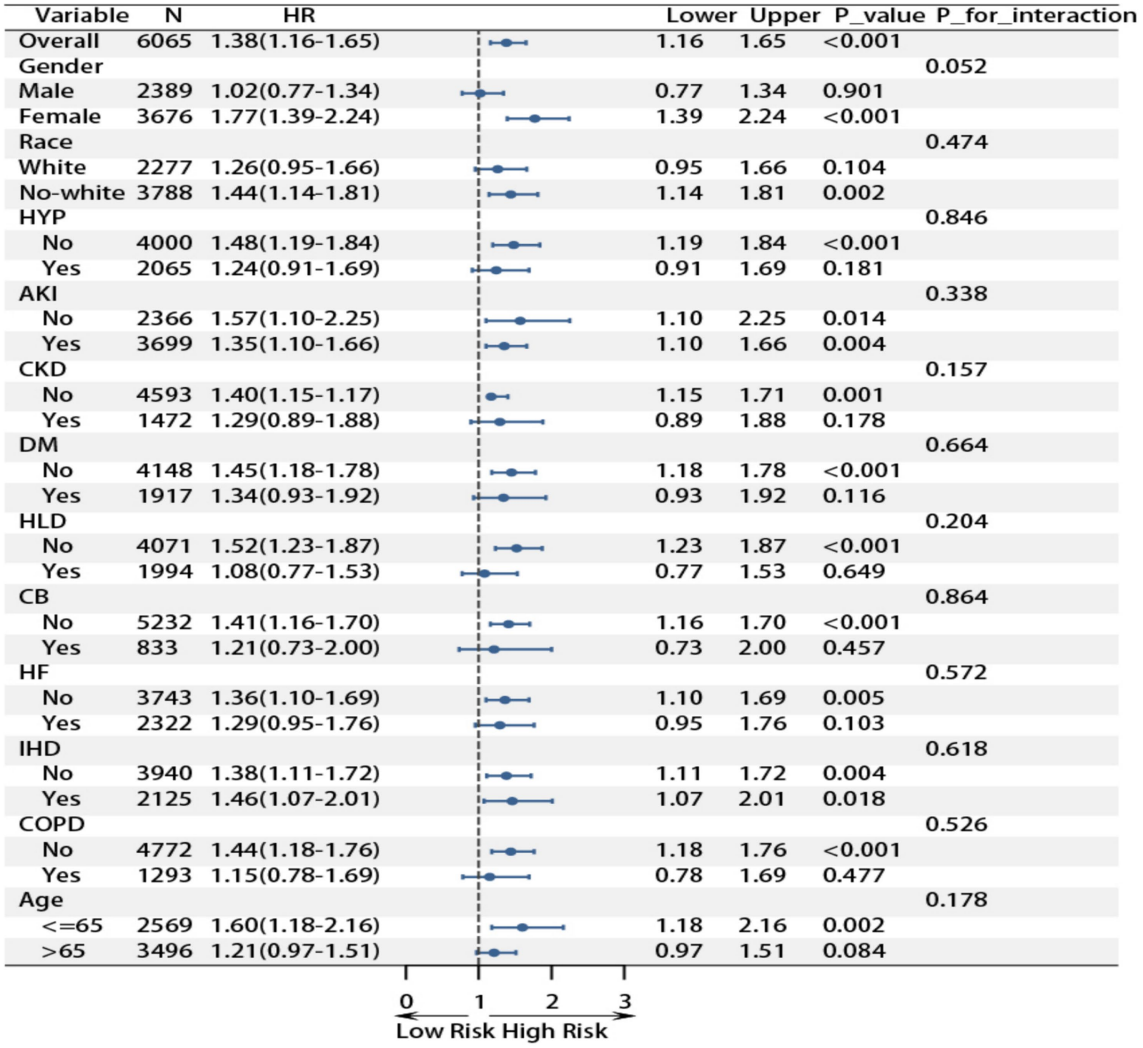
Subgroup analysis of RAR and 28 day hospital mortality rate.

Subgroup analyses demonstrated a consistent association between RAR and poor prognosis across most patient populations with pulmonary sepsis (Figures 2 and 3). For 28-day ICU mortality, each unit increase in RAR was associated with a 61% higher risk in the overall cohort (HR 1.61, 95% CI 1.36–1.91). This association remained significant in most subgroups and was particularly pronounced in females (HR 1.96, 95% CI 1.56–2.47), non-White individuals (HR 1.74, 95% CI 1.39–2.17), and patients aged ≤65 years (HR 1.87, 95% CI 1.39–2.51). A test for interaction indicated a trend toward a stronger effect in females, which approached statistical significance (P for interaction = 0.052). A similar pattern was observed for 28-day in-hospital mortality, with an overall hazard ratio of 1.38 (95% CI 1.16–1.65). The association was again strongest in females (HR 1.77, 95% CI 1.39–2.24), non-White individuals (HR 1.44, 95% CI 1.14–1.81), and those ≤65 years (HR 1.60, 95% CI 1.18–2.16). Conversely, the association was not statistically significant in patients with hyperlipidemia (*p* = 0.649), chronic bronchitis (*p* = 0.457), or COPD (*p* = 0.477). Importantly, none of the formal tests for interaction were statistically significant (all *p* > 0.05), indicating that the relationship between RAR and mortality is generally stable across different demographic and clinical subgroups. These findings reinforce the potential clinical utility of RAR as a prognostic biomarker in pulmonary sepsis, demonstrating consistent predictive performance in the vast majority of patient populations.

### The incremental effect of RAR

To evaluate the incremental predictive value of RAR for 28-day ICU mortality, we added it to established disease severity scores (APACHE II, APS III, SAPS II, OASIS, SIRS, and SOFA) and compared the area under the curve (AUC). As shown in Supplementary Figures S1A–F, incorporating RAR consistently enhanced the predictive performance of all scoring systems. The AUC improved for APACHE II (from 0.62 to 0.64), APS III (0.64–0.65), SAPS II (0.61–0.63), OASIS (0.64–0.66), SIRS (0.54–0.60), and SOFA (from 0.61–0.63).

### External cohort verification

**Figure 4:**
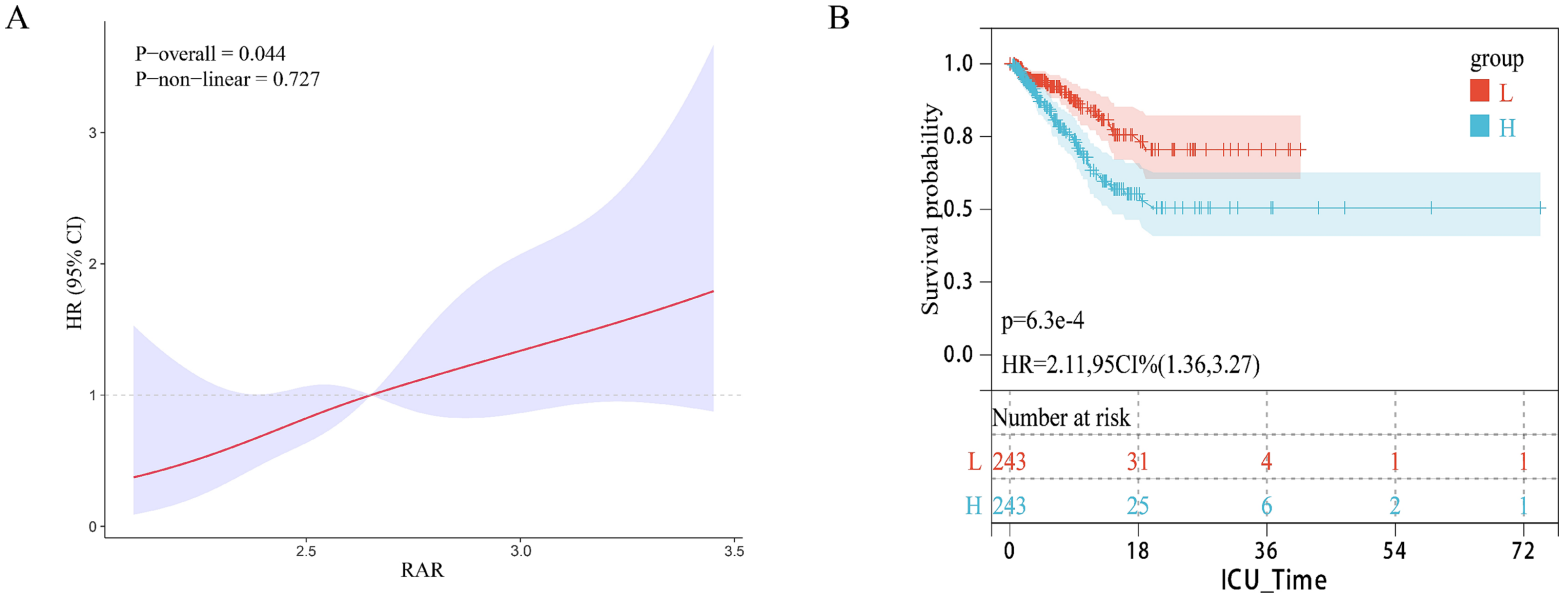
Association between RAR and 28-day survival in the external validation cohort. **(A)** Restricted cubic spline plot showing the dose–response relationship between continuous RAR values and 28-day in-hospital mortality in the external validation cohort. **(B)** Kaplan–Meier survival curves illustrate the probability of survival from ICU death 28 days, stratified by RAR score stratification.

The predictive value of RAR for 28-day ICU mortality was further validated in an external cohort of 486 patients with pulmonary sepsis, which had a 28-day mortality rate of 18.51%. As summarized in Table 3, in the unadjusted model (Model 1), each unit increase in RAR was associated with a 151% higher risk of ICU mortality (HR 2.51, 95% CI 1.47–4.29). When analyzed by tertiles, the Q3 group had a 134% increased risk (HR 2.34, 95% CI 1.37–4.01) compared to the Q1 group, while the risk increase in the Q2 group was not statistically significant (HR 1.56, 95% CI 0.89–2.76). This association persisted after multivariable adjustment. In the fully adjusted model (Model 3), each unit increase in RAR remained associated with a 99% increased risk (HR 1.99, 95% CI 1.09–3.66). Similarly, the Q3 group maintained a 79% higher risk (HR 1.79, 95% CI 1.02–3.14) compared to Q1, whereas the risk for Q2 remained non-significant. Although the hazard ratios in the validation cohort were somewhat attenuated compared to the derivation cohort, the overall dose–response pattern was consistent, with the highest risk (Q3) being statistically significant across all models. This dose–response relationship was confirmed by restricted cubic spline (RCS) analysis, which showed a significant, positive association (*p* = 0.044; Figure 4A). Kaplan–Meier survival curves provided further evidence, demonstrating that higher RAR scores were significantly associated with increased mortality (HR = 2.11, 95% CI: 1.36–3.27; Figure 4B).

**Table 3:** The relationship between RAR score and external verification cohort 28 day ICU mortality rate.

Characteristic	Model 1			Model 2			Model 3		
	HR	95% CI	*p*-value	HR	95% CI	*p*-value	HR	95% CI	*p*-value
RAR	2.51	1.47, 4.29	0.001	2.28	1.30, 3.99	<0.001	1.99	1.09, 3.66	0.026
RAR group									
Q1	Ref	Ref		Ref	Ref		Ref	Ref	
Q2	1.56	0.89, 2.76	0.121	1.57	0.89,2.77	0.119	1.35	0.75, 2.41	0.316
Q3	2.34	1.37, 4.01	0.002	2.14	1.24, 3.70	0.006	1.79	1.02, 3.14	0.044

CI, Confidence Interval; HR, Hazard Ratio; Ref, reference. Model 1: Unadjusted. Model 2: Adjusted for age, gender, Body Mass Index (BMI), and Chronic Obstructive Pulmonary Disease (COPD). Model 3: Adjusted for all variables in Model 2, plus: SOFA, APSIII, SAPSII, OASIS, APACHEII, TB, URE, CRRT, GC, and RDW.

### Construction and validation of risk prediction models related to RAR

**Figure 5:**
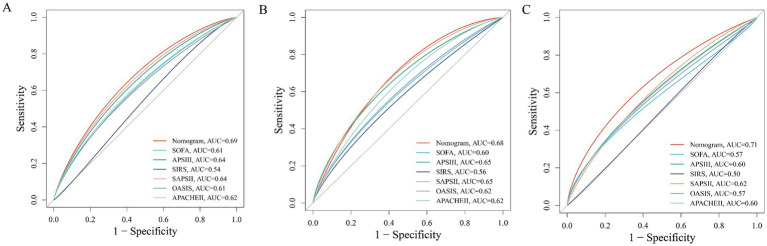
Receiver operating characteristic (ROC) curves comparing the predictive performance of the novel nomogram and conventional severity scores for 28-day mortality. **(A)** Test cohort 1; **(B)** train cohort 2; **(C)** external verification cohort.

**Figure 6:**
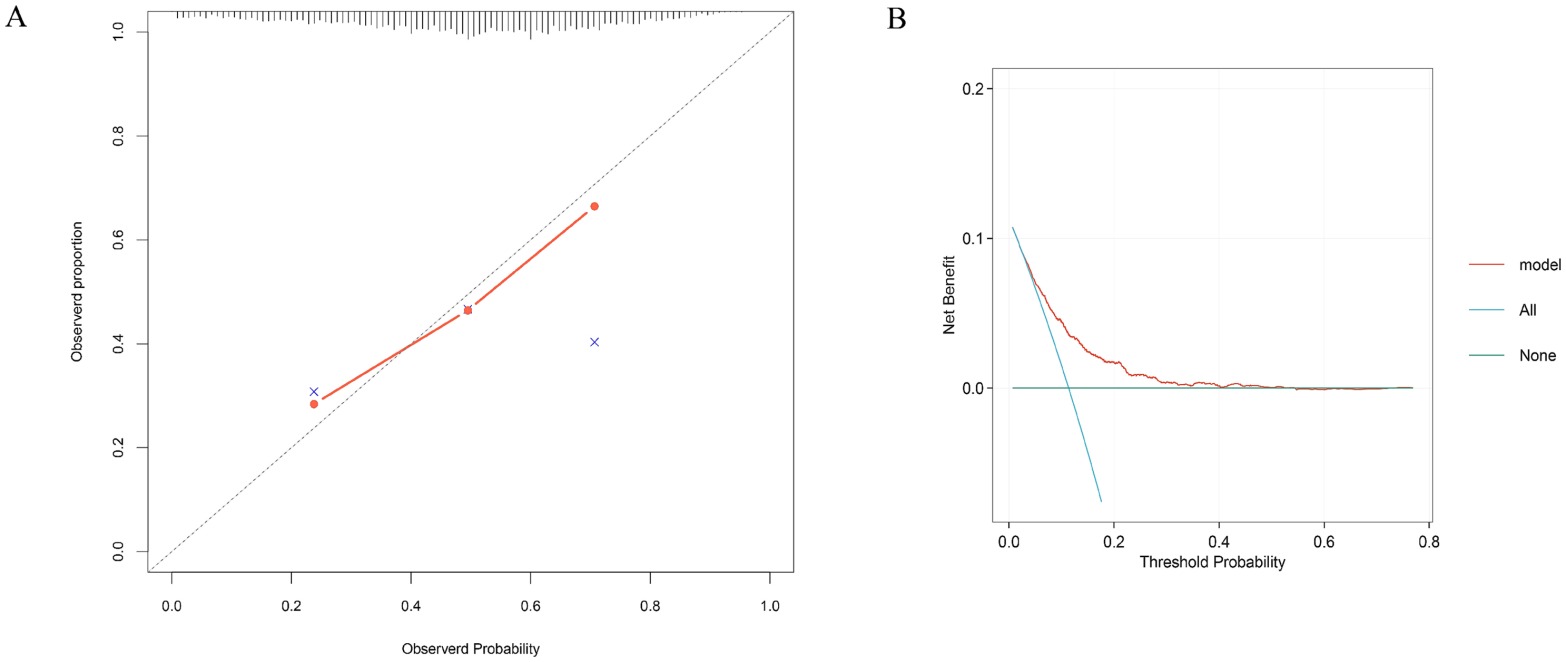
Calibration curve and decision curve of the training cohort.

The internal cohort was randomly split into a training set (70%) and a testing set (30%). Within the training set, we employed the Boruta algorithm (Supplementary Figure S3A), Random Survival Forests (RSF) (Supplementary Figure S3B), and LASSO regression (Figures 2A,B) to identify features associated with 28-day ICU mortality. Boruta identified variables associated with the binary occurrence of mortality, while RSF provided importance rankings that accounted for follow-up time and censoring. The intersection of variables identified by all three methods yielded nine candidate variables (Supplementary Figure S3C). Subsequent multivariable Cox regression confirmed that they were independent predictors: RAR, platelet count (PLT), partial pressure of oxygen (PO2), lymphocyte count, age, SAPS II, total bilirubin (TB), mechanical ventilation, and lactate dehydrogenase (LDH) (Supplementary Figure S4). The final risk prediction model for 28-day ICU mortality was constructed as follows: Risk Score = (0.588 × RAR) + (0.020 × Age) + (0.018 × SAPS II) + (0.027 × TB) + (−0.994 × Mechanical Ventilation) + (−0.001 × PLT) + (−0.002 × PO2) + (−0.012 × Lymphocyte count) + (0.000075 × LDH). Compared to traditional severity scores (SOFA, APS III, SAPS II, OASIS, SIRS, APACHE II), this model demonstrated superior sensitivity and specificity (Table 4). The model showed consistent discriminative ability across all cohorts, with AUCs of 0.69 in the internal training set (Figure 5A), 0.68 in the internal test set (Figure 5B), and 0.71 in the external validation cohort (Figure 5C). Furthermore, the calibration curve (Figure 6A) indicated excellent agreement between predicted and observed outcomes across the entire risk spectrum, with the plot closely aligning with the ideal 45-degree line. Decision curve analysis (Figure 6B) confirmed the model’s clinical utility, demonstrating a superior net benefit over default strategies across a wide range of threshold probabilities (approximately 5–70%).

**Table 4:** Final multivariable cox proportional hazards model for 28-day mortality risk prediction.

Variable	Coefficient (*β*)	Hazard ratio (HR)	Standard error (SE)	*Z*-value	*p*-value
RAR	0.588	1.801	0.098	5.981	<0.001
Age	0.020	1.020	0.003	7.634	<0.001
SAPSII	0.018	1.018	0.002	7.624	<0.001
TB	0.027	1.028	0.005	5.887	<0.001
Ventilation	−0.994	0.370	0.124	−8.004	<0.001
PLT	−0.001	0.999	0.000	−4.443	<0.001
PO2	−0.002	0.998	0.000	−5.024	<0.001
Lymphocyte count	−0.012	0.988	0.004	−3.341	0.001
LDH	7.51 × 10^−5^	1.000	2.43 × 10^−5^	3.086	0.002

RAR, Red cell distribution width to Albumin Ratio; SAPSII, Simplified Acute Physiology Score II; TB, Total Bilirubin; PLT, Platelet Count; PO2, Partial Pressure of Oxygen; Lymphocyte count, Lymphocyte Count; LDH, Lactate Dehydrogenase. This model was developed using an ensemble machine learning approach for feature selection (Boruta algorithm, LASSO regression, and Random Forests) followed by multivariable Cox regression.

## Discussion

This study provides a comprehensive, multi-faceted body of evidence that systematically establishes, for the first time, the core prognostic value of the Red cell Distribution Width to Albumin Ratio (RAR) in pulmonary sepsis. We not only demonstrated a strong, graded relationship and a dose–response dependency between RAR and both disease severity and clinical outcomes but also, through rigorous multivariable adjustment, extensive subgroup stability analyses, and independent external validation, confirmed its reliability as an independent prognostic biomarker. These findings deepen our understanding of the pathophysiology of sepsis and offer a novel theoretical framework and a practical tool for risk stratification in critical care medicine.

The superior predictive performance of RAR stems from its deep integration of core pathophysiological processes in sepsis. Unlike traditional, unidimensional biomarkers, RAR elegantly combines RDW—a marker of inflammatory stress—with albumin, which reflects nutritional reserve and inflammatory control, thereby forming a unique quantitative indicator of the “inflammation-nutrition-oxidative stress triad” (25, 26). An elevated RDW is not merely increased red cell size heterogeneity; rather, it represents the final common pathway of multiple mechanisms, including disrupted erythropoiesis by inflammatory cytokines (e.g., IL-6, TNF-*α*), oxidative damage to red cell membranes, and erythropoietin resistance (27, 28). Concurrently, hypoalbuminemia depicts a complementary pathophysiological landscape: systemic inflammation drives capillary leakage, repurposes hepatic synthesis (favoring acute-phase proteins over albumin), and promotes a hypercatabolic state, collectively leading to albumin depletion (29, 30). Consequently, an elevated RAR precisely captures the most perilous pathological state—one of “high inflammatory stress” coupled with “low physiological reserve.” This likely explains why RAR is a more sensitive predictor of mortality than either of its individual components, offering a novel biological perspective for understanding the intrinsic vulnerability of septic patients.

Furthermore, our multi-level analyses confirm the reliability and generalizability of RAR’s predictive value. Subgroup and interaction analyses demonstrated that the association between RAR and mortality was consistent across the vast majority of pre-specified subgroups, with no significant effect modifications detected. It is noteworthy that this association was even more pronounced in females, non-White individuals, and patients aged ≤65 years, providing intriguing clues for future research into personalized risk assessment and the underlying biological mechanisms. Crucially, we successfully replicated our primary findings in an independent external validation cohort. Although the point estimates of the hazard ratios varied slightly due to population differences, the significant positive association between RAR and mortality, along with the dose–response trend, was consistently reproduced. This successful external validation substantially strengthens the robustness and generalizability of our conclusions.

From a clinical translation perspective, our findings hold a dual significance. First, as a parameter derived from routine, low-cost laboratory tests, RAR is easily accessible and calculable in diverse healthcare settings, making it highly suitable for early risk stratification to identify high-risk patients who may benefit from more intensive monitoring and aggressive intervention. Second, incremental model analysis demonstrated that incorporating RAR into established critical illness scores (e.g., APACHE II, SOFA) resulted in a clinically meaningful improvement in predictive performance, as measured by the area under the curve (AUC). This confirms that RAR provides unique prognostic information not captured by traditional scoring systems. Furthermore, the multivariate model we developed, which integrates RAR with other independent predictors, demonstrated robust and excellent predictive accuracy (AUC 0.68–0.71) across both internal and external validation cohorts, laying a solid foundation for a novel prognostic tool for pulmonary sepsis.

Not only that, our findings are further supported by recent evidence from COVID-19 research, where systemic inflammation indices have demonstrated similar prognostic utility. Pluta et al. reported that in critically ill COVID-19 patients admitted to the ICU, RDW on admission predicted death with an AUC of 0.72 (95% CI: 0.60–0.82; cut-off > 44.7 fL), closely mirroring the discriminatory performance of RAR observed in our study (31). Similarly, Aydınyılmaz et al. found that RDW predicted mortality in COVID-19 patients with an AUC of 0.75, and Sarkar S et al. demonstrated that RDW was among the most predictive complete blood count parameters for COVID-19 outcomes (32, 33). These consistent findings across different pneumonia etiologies—bacterial, viral (including SARS-CoV-2)—reinforce the robustness of RDW-based biomarkers, including RAR, as prognostic tools in critically ill patients with pulmonary infections. The convergence of evidence suggests that the pathophysiological mechanisms linking elevated RDW to adverse outcomes (i.e., inflammation, oxidative stress, and impaired erythropoiesis) are common across diverse infectious insults to the lung.

Despite establishing the prognostic value of RAR through rigorous multi-center validation, our study has several inherent limitations. First, its retrospective design, despite extensive multivariable adjustment, cannot entirely rule out residual confounding from unmeasured variables, such as palliative care decisions or pathogen virulence, which may introduce bias. Second, the reliance on a single, static measurement of RAR fails to capture its dynamic trajectory over time, which may hold richer prognostic information than a baseline value alone. Furthermore, while external validation enhances the reliability of our findings, all data originated from a similar healthcare system; the generalizability of RAR requires confirmation in prospective cohorts from diverse healthcare settings. Third, we did not perform post-hoc power calculations for the observed effects, as such calculations are conceptually circular and not recommended for interpreting findings from completed studies. Instead, we assessed the precision of our estimates through confidence intervals and confirmed model stability through events-per-variable (EPV) ratios. The primary MIMIC-IV cohort had an EPV of approximately 30.3 for ICU mortality, far exceeding the recommended minimum of 10, indicating adequate statistical power and stable effect estimates. Fourth, while external validation enhances the reliability of our findings, all data originated from a similar healthcare system; the generalizability of RAR requires confirmation in prospective cohorts from diverse healthcare settings. Fifth, while the primary MIMIC-IV cohort had a robust events per variable ratio (EPV > 20), the external validation cohort had a smaller sample size and a lower EPV (approximately 6.4), which may affect the stability of effect estimates in this subset. Sixth, while our incremental analysis focused on AUC as a measure of discriminative ability, we acknowledge that a more comprehensive evaluation—including metrics such as the net reclassification improvement (NRI), integrated discrimination improvement (IDI), and decision curve analysis (DCA)—would provide additional insights into the clinical utility of RAR. Future studies specifically designed for prediction model development should consider these metrics to fully characterize the added value of RAR in risk stratification. The most critical limitation is that our study does not establish a causal link between RAR and clinical outcomes. Whether actively lowering RAR through specific interventions directly improves patient survival remains a pivotal, unanswered question. These limitations, however, clearly delineate a path for future research: conducting large, multicenter prospective studies to define optimal RAR thresholds, investigating the clinical utility of its dynamic monitoring, and elucidating its precise pathophysiological role in sepsis to ultimately translate risk prediction into targeted therapeutic intervention.

## Conclusion

In conclusion, this study provides compelling evidence that the Red cell Distribution Width to Albumin Ratio (RAR) is an independent and reliable predictor of short-term mortality in patients with pulmonary sepsis. RAR is strongly associated with disease severity and adverse clinical outcomes, and its prognostic value has been consistently validated across diverse patient subgroups and in an independent external cohort. The integration of this readily available and inexpensive biomarker into clinical decision-support systems holds significant promise for improving risk stratification, thereby facilitating the optimal allocation of resources and enabling more personalized treatment. Prospective studies are now warranted to confirm these findings and to determine whether RAR-guided management strategies can ultimately improve patient outcomes.

## Data Availability

The datasets presented in this study can be found in online repositories. The names of the repository/repositories and accession number(s) can be found in the article/Supplementary material.
