# Supplementary material for: The red cell distribution width to albumin ratio as a novel biomarker for predicting short-term mortality in severe pulmonary sepsis: a retrospective study with dual-cohort validation
Source: Front Med (Lausanne). 2026 Apr 7;13:1805614. doi: 10.3389/fmed.2026.1805614 (PMC13096080; doi:10.3389/fmed.2026.1805614)
Supplement: Supplementary file 1 [file Supplementary_file_1.pdf]

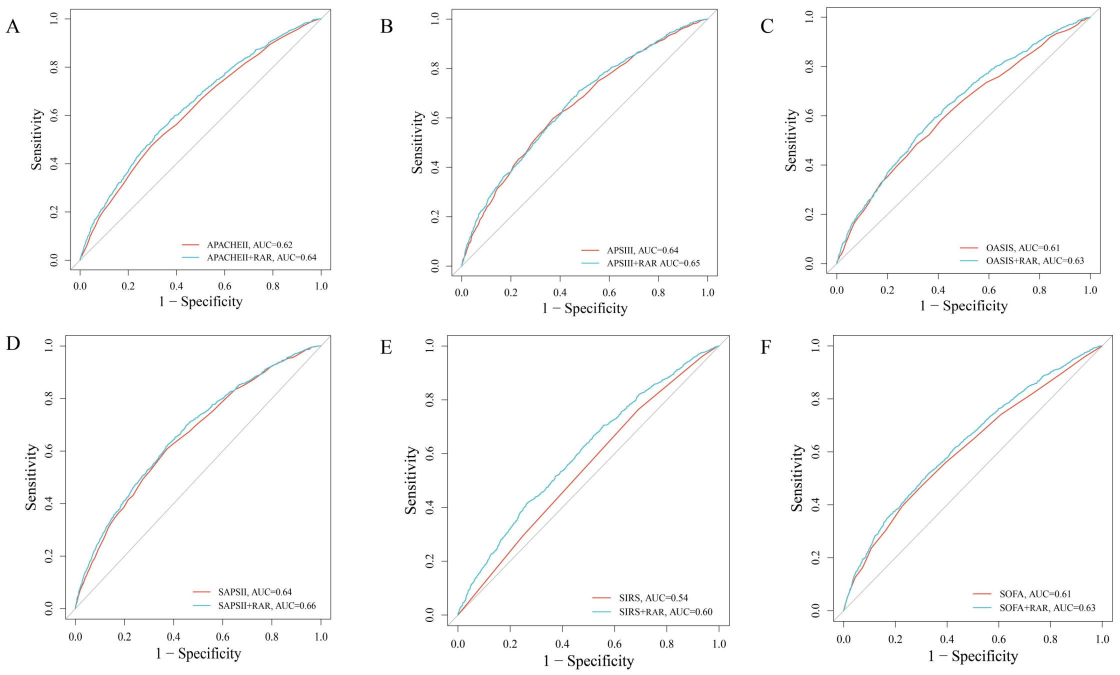

Figure S1: Incremental prognostic value of adding RAR to conventional severity scores for predicting 28-day in-ICU mortality.

Note: (A-F) Receiver operating characteristic (ROC) curves demonstrate the improvement in predictive performance when the RAR is added to five conventional severity scores: (A) APACHE II, (B) APS III, (C) SAPS II, (D) OASIS, (E) SIRS and (F) SOFA. Each panel compares the AUC of the original severity score (red) against the combined model of the severity score plus GNRI (blue) in the internal cohort. The specific AUC values for each model are provided in the main text.

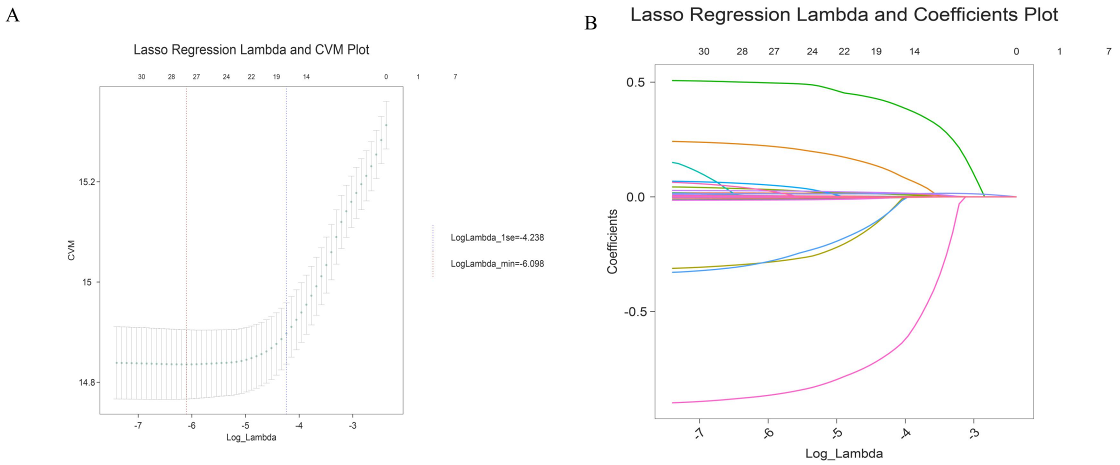

Figure S2: Variable selection using the Least Absolute Shrinkage and Selection Operator (LASSO) regression of factors associated with 28-day ICU mortality.

Note: (A) LASSO coefficient profiles of the candidate prognostic variables. Each curve represents

the coefficient path of a variable as the penalty parameter (λ) increases.(B) Ten-fold cross-validation for tuning parameter (λ) selection in the LASSO model. The left vertical dashed line (lambda.min) is drawn at the value of λ that gives the minimum mean cross-validated error. The right vertical dashed line (lambda.1se) is drawn at the largest value of λ such that the error is within one standard error of the minimum, which was used for feature selection.

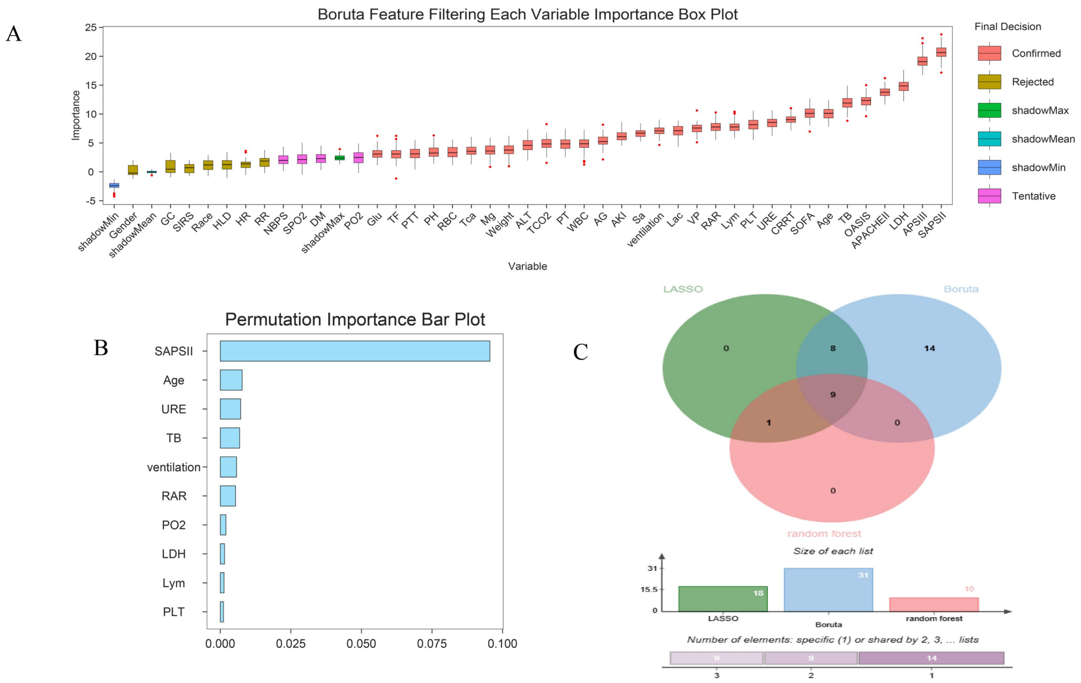

Figure S3: The screening process of feature variables

Note: (A):Boruta feature ranking algorithm, (B):Random Forest; (C):The Venn diagram shows the overlap of candidate predictor variables identified as important in three feature selection methods (Boruta algorithm, LASSO-COX regression random forest machine learning algorithm).

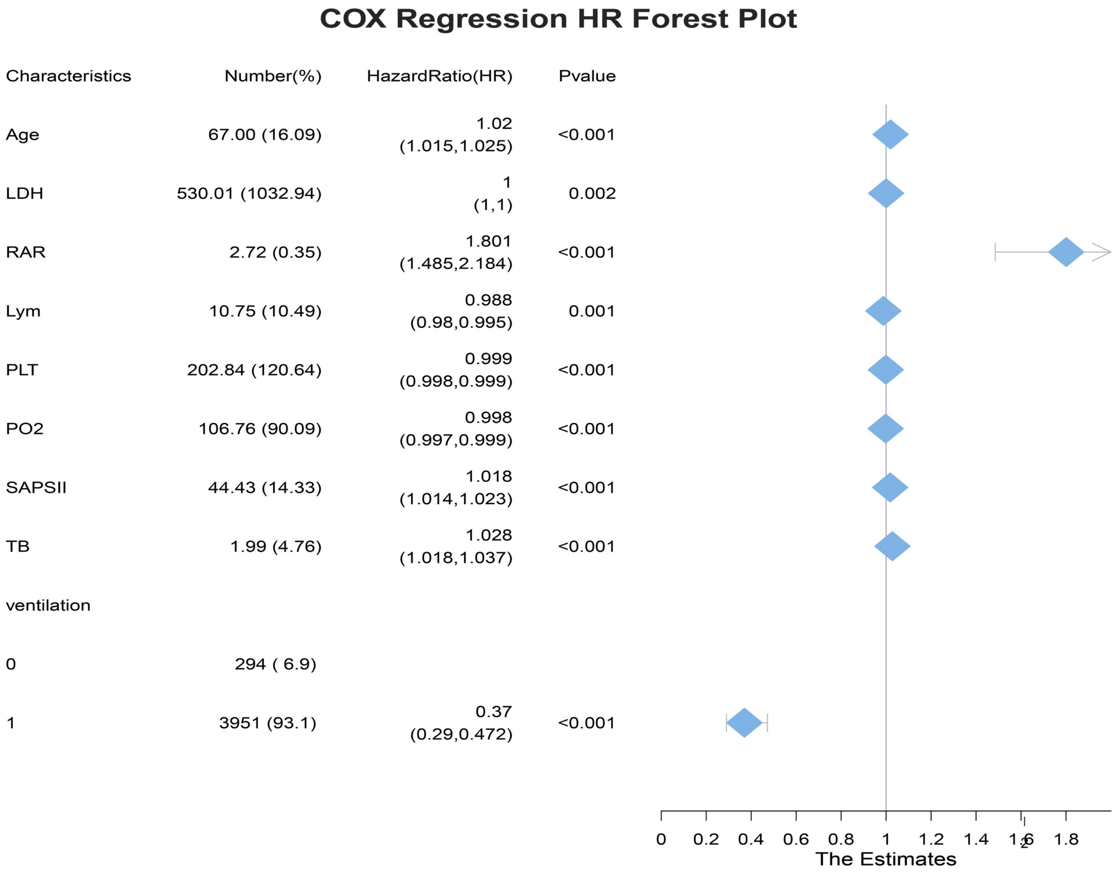

Figure S4: Multi factor COX analysis forest plot of 9 important variables selected in the final screening.

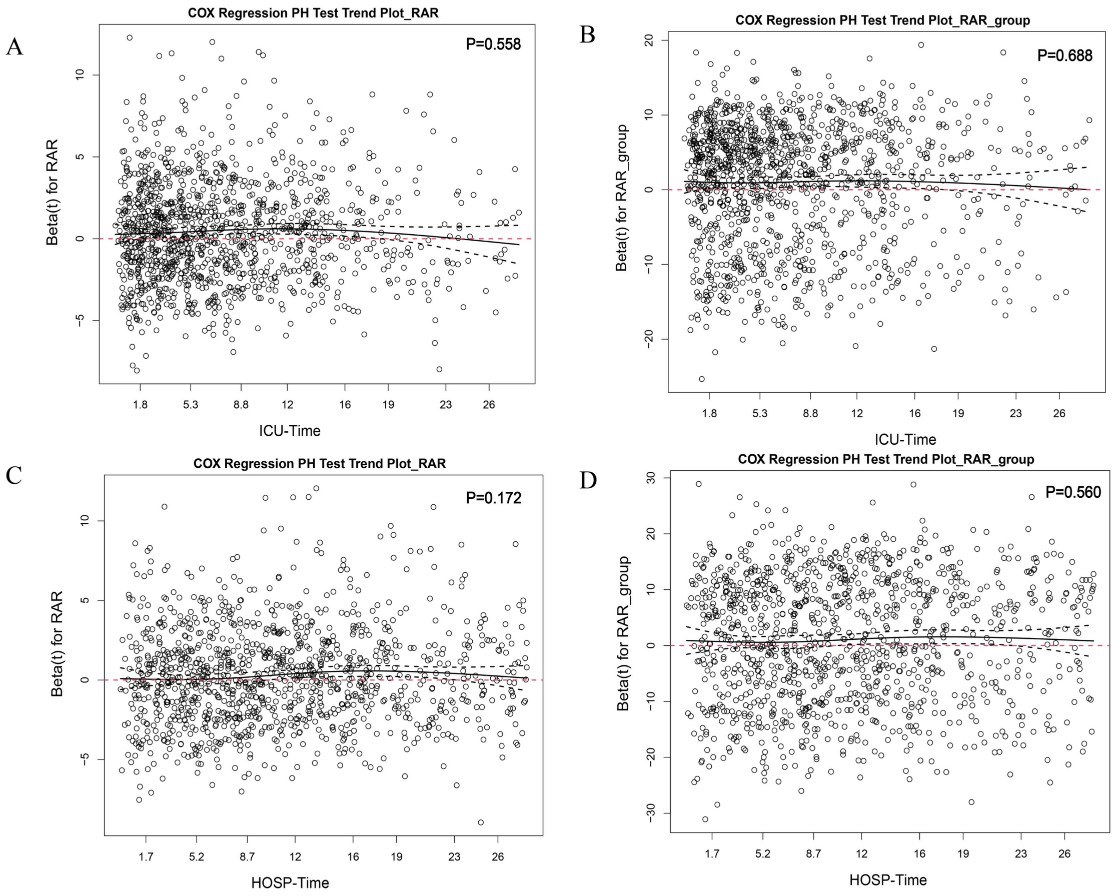

Figure S5: COX Regression PH Test Trend Plot

Note: (A, B) RAR as continuous and categorical (Q) variables in the 28 day ICU mortality proportion risk assessment trend chart.(C, D) RAR as continuous and categorical (Q) variables in the 28 day hospital mortality proportion risk assessment trend chart.

TableS1: Summary descriptives table by groups of 28day in-ICU mortality rate

Variable	[ALL] *N=6065*	Survivor *N=4822*	No-survivor *N=1243*	P-value
RAR	2.72 (0.35)	2.69 (0.34)	2.82 (0.37)	<0.001
RAR.group	.	.	.	.
Age	67.0 (16.0)	66.2 (16.2)	69.8 (14.7)	<0.001
Gender:				0.196
M	2389 (39.4%)	1879 (39.0%)	510 (41.0%)	
F	3676 (60.6%)	2943 (61.0%)	733 (59.0%)	
Race:				0.004
No-white	2277 (37.5%)	1766 (36.6%)	511 (41.1%)	
White	3788 (62.5%)	3056 (63.4%)	732 (58.9%)	
Weight (kg)	83.0 (25.8)	83.6 (25.9)	80.5 (25.0)	<0.001
HYP:				0.432
No	4000 (66.0%)	3168 (65.7%)	832 (66.9%)	
Yes	2065 (34.0%)	1654 (34.3%)	411 (33.1%)	

Variable	[ALL] *N=6065*	Survivor *N=4822*	No-survivor *N=1243*	P-value
AKI:				<0.001
No	2366 (39.0%)	2046 (42.4%)	320 (25.7%)	
Yes	3699 (61.0%)	2776 (57.6%)	923 (74.3%)	
CKD:				0.834
No	4593 (75.7%)	3655 (75.8%)	938 (75.5%)	
Yes	1472 (24.3%)	1167 (24.2%)	305 (24.5%)	
DM:				0.001
No	4148 (68.4%)	3247 (67.3%)	901 (72.5%)	
Yes	1917 (31.6%)	1575 (32.7%)	342 (27.5%)	
HLD:				0.012
No	4071 (67.1%)	3199 (66.3%)	872 (70.2%)	
Yes	1994 (32.9%)	1623 (33.7%)	371 (29.8%)	
CB:				0.145
No	5232 (86.3%)	4176 (86.6%)	1056 (85.0%)	

Variable	[ALL] *N=6065*	Survivor *N=4822*	No-survivor *N=1243*	P-value
Yes	833 (13.7%)	646 (13.4%)	187 (15.0%)	
HF:				0.143
No	3743 (61.7%)	2953 (61.2%)	790 (63.6%)	
Yes	2322 (38.3%)	1869 (38.8%)	453 (36.4%)	
IHD:				0.257
No	3940 (65.0%)	3115 (64.6%)	825 (66.4%)	
Yes	2125 (35.0%)	1707 (35.4%)	418 (33.6%)	
COPD:				0.095
No	4772 (78.7%)	3816 (79.1%)	956 (76.9%)	
Yes	1293 (21.3%)	1006 (20.9%)	287 (23.1%)	
SOFA	7.31 (3.60)	6.99 (3.42)	8.53 (4.00)	<0.001
APSIII	58.3 (22.0)	55.9 (20.7)	67.7 (24.3)	<0.001
SIRS	2.90 (0.86)	2.87 (0.87)	3.02 (0.80)	<0.001
SAPSII	44.4 (14.4)	42.7 (13.6)	50.8 (15.2)	<0.001

Variable	[ALL] *N=6065*	Survivor *N=4822*	No-survivor *N=1243*	P-value
OASIS	36.5 (8.47)	35.8 (8.24)	39.3 (8.78)	<0.001
APACHEII	21.8 (7.31)	21.2 (7.09)	24.4 (7.56)	<0.001
HR (beats/min)	93.9 (21.8)	93.4 (21.7)	95.4 (21.9)	0.004
NBPS (mmHg)	120 (25.3)	121 (25.3)	119 (25.3)	0.011
NBPD (mmHg)	70.1 (89.4)	70.6 (99.8)	67.9 (20.3)	0.081
RR (insp/min)	21.4 (6.97)	21.2 (6.83)	21.9 (7.45)	0.003
SpO_2 (%)	96.1 (12.1)	96.3 (13.3)	95.5 (4.96)	0.001
TF (°F)	98.2 (4.10)	98.3 (3.67)	97.8 (5.43)	0.003
HCT (%)	32.1 (7.19)	32.3 (7.13)	31.7 (7.40)	0.013
Hb (g/dL)	10.4 (2.38)	10.4 (2.38)	10.2 (2.40)	0.004
PLT (K/μL)	204 (121)	208 (120)	190 (123)	<0.001
RDW (%)	15.9 (2.65)	15.8 (2.58)	16.4 (2.83)	<0.001
RBC (m/uL)	3.50 (0.84)	3.52 (0.83)	3.41 (0.85)	<0.001
WBC (K/μL)	13.9 (12.3)	13.6 (12.0)	15.3 (13.4)	<0.001

Variable	[ALL] *N=6065*	Survivor *N=4822*	No-survivor *N=1243*	P-value
ALB (g/dL)	2.90 (0.61)	2.93 (0.59)	2.77 (0.63)	<0.001
AG (mEq/L)	15.4 (4.94)	15.2 (4.83)	16.0 (5.31)	<0.001
Tca (mg/dL)	8.22 (0.96)	8.24 (0.94)	8.17 (1.02)	0.026
Cl (mEq/L)	103 (7.73)	103 (7.59)	103 (8.28)	0.562
Glu (mg/dL)	154 (85.2)	152 (79.1)	160 (105)	0.024
K (mEq/L)	4.27 (0.82)	4.26 (0.81)	4.31 (0.85)	0.095
Na (mEq/L)	138 (6.46)	138 (6.31)	138 (7.02)	0.864
TCO_2 (mEq/L)	24.8 (6.37)	25.0 (6.29)	24.0 (6.60)	<0.001
Lac (mmol/L)	2.44 (2.13)	2.32 (1.98)	2.92 (2.60)	<0.001
PCO_2 (mmHg)	44.7 (14.0)	44.6 (13.8)	44.9 (14.8)	0.610
PH (pH units)	7.34 (0.11)	7.35 (0.11)	7.33 (0.12)	<0.001
PO_2 (mmHg)	107 (90.1)	109 (91.6)	98.8 (83.3)	<0.001
INR (ratio)	1.67 (1.12)	1.64 (1.09)	1.82 (1.20)	<0.001
PT (seconds)	18.1 (11.2)	17.7 (10.8)	19.7 (12.5)	<0.001

Variable	[ALL] *N=6065*	Survivor *N=4822*	No-survivor *N=1243*	P-value
PTT (seconds)	39.9 (24.3)	39.0 (23.4)	43.2 (27.3)	<0.001
ALT (IU/L)	135 (552)	127 (529)	167 (633)	0.037
AST (IU/L)	221 (911)	206 (867)	283 (1065)	0.018
TB (mg/dL)	1.92 (4.54)	1.67 (3.97)	2.89 (6.18)	<0.001
CRE (mg/dL)	1.78 (1.79)	1.77 (1.85)	1.81 (1.50)	0.422
URE (mg/dL)	34.2 (26.3)	32.9 (25.5)	39.4 (28.8)	<0.001
LDH (U/L)	537 (1082)	487 (932)	727 (1516)	<0.001
Mg (mg/dL)	2.00 (0.47)	1.99 (0.47)	2.04 (0.47)	0.001
Lymphocyte count (%)	10.7 (10.3)	10.9 (10.2)	9.54 (10.6)	<0.001
CRRT:				<0.001
No	5301 (87.4%)	4316 (89.5%)	985 (79.2%)	
Yes	764 (12.6%)	506 (10.5%)	258 (20.8%)	
ventilation:				0.024

Variable	[ALL] *N=6065*	Survivor *N=4822*	No-survivor *N=1243*	P-value
No	426 (7.02%)	320 (6.64%)	106 (8.53%)	
Yes	5639 (93.0%)	4502 (93.4%)	1137 (91.5%)	
Sa:				<0.001
No	1357 (22.4%)	1170 (24.3%)	187 (15.0%)	
Yes	4708 (77.6%)	3652 (75.7%)	1056 (85.0%)	
GC:				0.014
No	3472 (57.2%)	2799 (58.0%)	673 (54.1%)	
Yes	2593 (42.8%)	2023 (42.0%)	570 (45.9%)	
VP:				<0.001
No	1866 (30.8%)	1649 (34.2%)	217 (17.5%)	
Yes	4199 (69.2%)	3173 (65.8%)	1026 (82.5%)	
ABX:				0.588
No	4 (0.07%)	4 (0.08%)	0 (0.00%)	
Yes	6061 (99.9%)	4818 (99.9%)	1243 (100%)	

Variable	[ALL] *N=6065*	Survivor *N=4822*	No-survivor *N=1243*	P-value
Hosp_Time	18.9 (18.3)	20.5 (19.3)	12.8 (11.3)	<0.001
Hosp_dead	0.19 (0.39)	0.00 (0.00)	0.94 (0.24)	0.000
ICU_Time	8.12 (9.01)	8.23 (9.64)	7.70 (5.95)	0.014

Table S2: Summary descriptives table by groups of 28day in-hospital mortality rate

Variable	[ALL] *N=6065*	Survivor *N=4895*	No-survivor *N=1170*	P-value
RAR	2.72 (0.35)	2.69 (0.34)	2.82 (0.38)	<0.001
RAR.group	.	.	.	.
Age	67.0 (16.0)	66.2 (16.2)	70.2 (14.6)	<0.001
Gender:				0.056
No	2389 (39.4%)	1899 (38.8%)	490 (41.9%)	
Yes	3676 (60.6%)	2996 (61.2%)	680 (58.1%)	
Race:				0.001

Variable	[ALL] *N=6065*	Survivor *N=4895*	No-survivor *N=1170*	P-value
No	2277 (37.5%)	1787 (36.5%)	490 (41.9%)	
Yes	3788 (62.5%)	3108 (63.5%)	680 (58.1%)	
Weight (kg)	83.0 (25.8)	83.5 (25.8)	80.6 (25.4)	<0.001
HYP:				0.543
No	4000 (66.0%)	3219 (65.8%)	781 (66.8%)	
Yes	2065 (34.0%)	1676 (34.2%)	389 (33.2%)	
AKI:				<0.001
No	2366 (39.0%)	2056 (42.0%)	310 (26.5%)	
Yes	3699 (61.0%)	2839 (58.0%)	860 (73.5%)	
CKD:				0.907
No	4593 (75.7%)	3709 (75.8%)	884 (75.6%)	
Yes	1472 (24.3%)	1186 (24.2%)	286 (24.4%)	
DM:				0.002
No	4148 (68.4%)	3303 (67.5%)	845 (72.2%)	

Variable	[ALL] *N=6065*	Survivor *N=4895*	No-survivor *N=1170*	P-value
Yes	1917 (31.6%)	1592 (32.5%)	325 (27.8%)	
HLD:				0.022
No	4071 (67.1%)	3252 (66.4%)	819 (70.0%)	
Yes	1994 (32.9%)	1643 (33.6%)	351 (30.0%)	
CB:				0.075
No	5232 (86.3%)	4242 (86.7%)	990 (84.6%)	
Yes	833 (13.7%)	653 (13.3%)	180 (15.4%)	
HF:				0.193
No	3743 (61.7%)	3001 (61.3%)	742 (63.4%)	
Yes	2322 (38.3%)	1894 (38.7%)	428 (36.6%)	
IHD:				0.325
No	3940 (65.0%)	3165 (64.7%)	775 (66.2%)	
Yes	2125 (35.0%)	1730 (35.3%)	395 (33.8%)	
COPD:				0.032

Variable	[ALL] *N=6065*	Survivor *N=4895*	No-survivor *N=1170*	P-value
No	4772 (78.7%)	3879 (79.2%)	893 (76.3%)	
Yes	1293 (21.3%)	1016 (20.8%)	277 (23.7%)	
SOFA	7.31 (3.60)	7.01 (3.43)	8.56 (4.00)	<0.001
APSIII	58.3 (22.0)	56.0 (20.7)	68.0 (24.5)	<0.001
SIRS	2.90 (0.86)	2.87 (0.87)	3.02 (0.80)	<0.001
SAPSII	44.4 (14.4)	42.8 (13.7)	50.9 (15.2)	<0.001
OASIS	36.5 (8.47)	35.8 (8.25)	39.5 (8.72)	<0.001
APACHEII	21.8 (7.31)	21.2 (7.10)	24.4 (7.59)	<0.001
HR (beats/min)	93.9 (21.8)	93.6 (21.7)	95.0 (21.8)	0.038
NBPS (mmHg)	120 (25.3)	121 (25.3)	119 (25.5)	0.016
NBPD (mmHg)	70.1 (89.4)	70.6 (99.0)	67.8 (20.2)	0.070
RR (insp/min)	21.4 (6.97)	21.3 (6.83)	21.9 (7.50)	0.009
SpO_2 (%)	96.1 (12.1)	96.3 (13.2)	95.4 (5.04)	<0.001
TF (°F)	98.2 (4.10)	98.3 (3.64)	97.8 (5.58)	0.003

Variable	[ALL] *N=6065*	Survivor *N=4895*	No-survivor *N=1170*	P-value
HCT (%)	32.1 (7.19)	32.2 (7.15)	31.9 (7.37)	0.234
Hb (g/dL)	10.4 (2.38)	10.4 (2.38)	10.3 (2.40)	0.089
PLT (K/μL)	204 (121)	207 (120)	191 (123)	<0.001
RDW (%)	15.9 (2.65)	15.8 (2.60)	16.4 (2.80)	<0.001
RBC (m/uL)	3.50 (0.84)	3.51 (0.84)	3.43 (0.85)	0.003
WBC (K/μL)	13.9 (12.3)	13.6 (12.3)	15.3 (12.3)	<0.001
ALB (g/dL)	2.90 (0.61)	2.93 (0.60)	2.76 (0.63)	<0.001
AG (mEq/L)	15.4 (4.94)	15.2 (4.84)	16.0 (5.32)	<0.001
Tca (mg/dL)	8.22 (0.96)	8.24 (0.95)	8.15 (0.99)	0.004
Cl (mEq/L)	103 (7.73)	103 (7.59)	103 (8.30)	0.608
Glu (mg/dL)	154 (85.2)	152 (79.0)	160 (107)	0.020
K (mEq/L)	4.27 (0.82)	4.26 (0.81)	4.32 (0.86)	0.062
Na (mEq/L)	138 (6.46)	138 (6.31)	138 (7.07)	0.814
TCO_2 (mEq/L)	24.8 (6.37)	25.0 (6.28)	24.1 (6.67)	<0.001

Variable	[ALL] *N=6065*	Survivor *N=4895*	No-survivor *N=1170*	P-value
Lac (mmol/L)	2.44 (2.13)	2.32 (1.97)	2.96 (2.64)	<0.001
PCO_2 (mmHg)	44.7 (14.0)	44.6 (13.8)	45.1 (15.0)	0.271
PH (pH units)	7.34 (0.11)	7.35 (0.11)	7.33 (0.12)	<0.001
PO_2 (mmHg)	107 (90.1)	109 (91.8)	97.8 (81.9)	<0.001
INR (ratio)	1.67 (1.12)	1.64 (1.10)	1.82 (1.19)	<0.001
PT (seconds)	18.1 (11.2)	17.8 (10.9)	19.7 (12.3)	<0.001
PTT (seconds)	39.9 (24.3)	39.1 (23.5)	43.2 (27.3)	<0.001
ALT (IU/L)	135 (552)	125 (525)	176 (652)	0.014
AST (IU/L)	221 (911)	204 (861)	296 (1096)	0.007
TB (mg/dL)	1.92 (4.54)	1.72 (4.10)	2.78 (5.96)	<0.001
CRE (mg/dL)	1.78 (1.79)	1.77 (1.85)	1.81 (1.50)	0.493
URE (mg/dL)	34.2 (26.3)	32.9 (25.5)	39.5 (28.9)	<0.001
LDH (U/L)	537 (1082)	485 (926)	752 (1559)	<0.001
Mg (mg/dL)	2.00 (0.47)	1.99 (0.47)	2.04 (0.47)	0.001

Variable	[ALL] *N=6065*	Survivor *N=4895*	No-survivor *N=1170*	P-value
Lymphocyte count (%)	10.7 (10.3)	11.0 (10.4)	9.29 (9.80)	<0.001
CRRT:				<0.001
No	5301 (87.4%)	4374 (89.4%)	927 (79.2%)	
Yes	764 (12.6%)	521 (10.6%)	243 (20.8%)	
ventilation:				0.051
No	426 (7.02%)	328 (6.70%)	98 (8.38%)	
Yes	5639 (93.0%)	4567 (93.3%)	1072 (91.6%)	
Sa:				<0.001
No	1357 (22.4%)	1182 (24.1%)	175 (15.0%)	
Yes	4708 (77.6%)	3713 (75.9%)	995 (85.0%)	
GC:				0.315
No	3472 (57.2%)	2818 (57.6%)	654 (55.9%)	
Yes	2593 (42.8%)	2077 (42.4%)	516 (44.1%)	

Variable	[ALL] *N=6065*	Survivor *N=4895*	No-survivor *N=1170*	P-value
VP:				<0.001
No	1866 (30.8%)	1661 (33.9%)	205 (17.5%)	
Yes	4199 (69.2%)	3234 (66.1%)	965 (82.5%)	
ABX:				1.000
No	4 (0.07%)	4 (0.08%)	0 (0.00%)	
Yes	6061 (99.9%)	4891 (99.9%)	1170 (100%)	
Hosp_Time	18.9 (18.3)	20.8 (19.6)	11.0 (7.08)	<0.001
ICU_Time	8.12 (9.01)	8.26 (9.62)	7.54 (5.76)	0.001
ICU_dead	0.20 (0.40)	0.01 (0.12)	1.00 (0.00)	0.000

Table S3: Summary descriptives table by groups of external verification cohort Dead

	[ALL]	Survivor	No-survivor	P-value

**Variable**	***N=486***	***N=396***	***N=90***	
RAR.group				0.017
Q1	162 (33.3%)	142 (35.9%)	20 (22.2%)	
Q2	162 (33.3%)	132 (33.3%)	30 (33.3%)	
Q3	162 (33.3%)	122 (30.8%)	40 (44.4%)	
RAR	2.69 (0.34)	2.67 (0.33)	2.80 (0.35)	0.002
Age	67.1 (16.1)	66.7 (16.3)	68.6 (15.0)	0.294
Gender	0.61 (0.49)	0.62 (0.49)	0.60 (0.49)	0.779
Race	0.65 (0.48)	0.66 (0.47)	0.60 (0.49)	0.303
BMI (kg/m^2)	30.4 (12.7)	30.0 (11.8)	32.2 (15.9)	0.224
HYP:				0.611
No	311 (64.0%)	256 (64.6%)	55 (61.1%)	
Yes	175 (36.0%)	140 (35.4%)	35 (38.9%)	
AKI:				0.081
No	199 (40.9%)	170 (42.9%)	29 (32.2%)	
Yes	287 (59.1%)	226 (57.1%)	61 (67.8%)	

Variable	[ALL] *N=486*	Survivor *N=396*	No-survivor *N=90*	P-value
CKD:				0.690
No	384 (79.0%)	311 (78.5%)	73 (81.1%)	
Yes	102 (21.0%)	85 (21.5%)	17 (18.9%)	
DM:				1.000
No	340 (70.0%)	277 (69.9%)	63 (70.0%)	
Yes	146 (30.0%)	119 (30.1%)	27 (30.0%)	
HLD:				0.554
No	334 (68.7%)	275 (69.4%)	59 (65.6%)	
Yes	152 (31.3%)	121 (30.6%)	31 (34.4%)	
CB:				0.435
No	410 (84.4%)	337 (85.1%)	73 (81.1%)	
Yes	76 (15.6%)	59 (14.9%)	17 (18.9%)	
HF:				0.139
No	310 (63.8%)	246 (62.1%)	64 (71.1%)	

Variable	[ALL] *N=486*	Survivor *N=396*	No-survivor *N=90*	P-value
Yes	176 (36.2%)	150 (37.9%)	26 (28.9%)	
IHD:				0.070
No	325 (66.9%)	257 (64.9%)	68 (75.6%)	
Yes	161 (33.1%)	139 (35.1%)	22 (24.4%)	
COPD:				0.037
No	368 (75.7%)	308 (77.8%)	60 (66.7%)	
Yes	118 (24.3%)	88 (22.2%)	30 (33.3%)	
SOFA	7.20 (3.64)	6.96 (3.40)	8.27 (4.43)	0.010
APSIII	57.5 (21.5)	55.8 (19.7)	65.5 (27.0)	0.002
SIRS	2.95 (0.86)	2.95 (0.87)	2.97 (0.85)	0.864
SAPSII	43.7 (14.0)	42.5 (13.5)	48.6 (15.3)	0.001
OASIS	36.5 (8.30)	36.0 (7.92)	38.7 (9.52)	0.014
APACHEII	21.7 (7.32)	21.1 (6.93)	24.1 (8.48)	0.002
HR	94.1 (22.3)	94.5 (22.5)	92.4 (21.4)	0.398

Variable	[ALL] *N=486*	Survivor *N=396*	No-survivor *N=90*	P-value
(beats/min)				
NBPS (mmHg)	121 (25.8)	121 (25.5)	120 (27.5)	0.886
NBPD (mmHg)	68.9 (19.9)	68.8 (19.1)	69.5 (23.4)	0.807
RR (insp/min)	21.5 (7.19)	21.2 (7.18)	22.4 (7.20)	0.173
SpO_2 (%)	96.0 (4.20)	96.1 (4.06)	95.3 (4.76)	0.158
TF (°F)	98.2 (3.24)	98.2 (3.52)	98.2 (1.52)	0.922
HCT (%)	32.1 (7.59)	32.3 (7.33)	31.2 (8.62)	0.292
Hb (g/dL)	10.4 (2.49)	10.4 (2.43)	10.0 (2.76)	0.174
PLT (K/μL)	204 (121)	208 (122)	188 (114)	0.158
RDW (%)	15.9 (2.62)	15.6 (2.27)	17.1 (3.56)	<0.001
RBC (m/uL)	3.48 (0.89)	3.50 (0.84)	3.41 (1.06)	0.469
WBC (K/μL)	13.6 (9.22)	13.3 (8.01)	14.6 (13.3)	0.373
ALB (g/dL)	2.95 (0.61)	2.96 (0.60)	2.90 (0.63)	0.430

Variable	[ALL] *N=486*	Survivor *N=396*	No-survivor *N=90*	P-value
AG (mEq/L)	15.3 (4.52)	15.3 (4.52)	15.5 (4.52)	0.712
Tca (mg/dL)	8.25 (0.99)	8.23 (0.97)	8.36 (1.09)	0.292
Cl (mEq/L)	104 (7.63)	104 (7.38)	104 (8.67)	0.852
Glu (mg/dL)	152 (69.8)	153 (70.5)	149 (66.5)	0.557
K (mEq/L)	4.30 (0.80)	4.27 (0.80)	4.40 (0.79)	0.159
Na (mEq/L)	139 (6.51)	139 (6.14)	139 (8.01)	0.803
TCO_2 (mEq/L)	24.8 (6.17)	24.8 (6.23)	24.7 (5.91)	0.864
Lac (mmol/L)	2.48 (2.18)	2.41 (2.20)	2.81 (2.08)	0.100
PCO_2 (mmHg)	44.4 (14.3)	44.1 (13.8)	45.4 (16.1)	0.485
PH (pH units)	7.35 (0.11)	7.35 (0.11)	7.34 (0.12)	0.500
PO_2 (mmHg)	106 (84.8)	109 (88.0)	92.2 (67.6)	0.055
INR (ratio)	1.75 (1.57)	1.76 (1.70)	1.72 (0.80)	0.734
PT (seconds)	18.7 (13.4)	18.7 (14.3)	18.7 (8.57)	0.985
PTT (seconds)	40.1 (23.7)	39.8 (23.6)	41.5 (24.3)	0.553

Variable	[ALL] *N=486*	Survivor *N=396*	No-survivor *N=90*	P-value
ALT (IU/L)	136 (410)	137 (417)	129 (382)	0.846
AST (IU/L)	251 (1015)	230 (838)	339 (1577)	0.529
TB (mg/dL)	2.06 (4.83)	1.75 (4.12)	3.41 (7.05)	0.034
CRE (mg/dL)	1.76 (1.77)	1.70 (1.65)	2.03 (2.21)	0.191
URE (mg/dL)	33.2 (25.5)	32.0 (23.7)	38.5 (31.9)	0.072
LDH (U/L)	551 (1082)	514 (1000)	715 (1382)	0.195
Mg (mg/dL)	1.98 (0.42)	1.97 (0.42)	2.01 (0.43)	0.487
Lymphocyte count (%)	10.1 (8.61)	10.4 (9.02)	8.58 (6.34)	0.026
CRRT:				<0.001
No	424 (87.2%)	357 (90.2%)	67 (74.4%)	
Yes	62 (12.8%)	39 (9.85%)	23 (25.6%)	
ventilation:				0.594
No	23 (4.73%)	18 (4.55%)	5 (5.56%)	

Variable	[ALL] *N=486*	Survivor *N=396*	No-survivor *N=90*	P-value
Yes	463 (95.3%)	378 (95.5%)	85 (94.4%)	
Sa:				0.847
No	107 (22.0%)	86 (21.7%)	21 (23.3%)	
Yes	379 (78.0%)	310 (78.3%)	69 (76.7%)	
GC:				0.010
No	283 (58.2%)	242 (61.1%)	41 (45.6%)	
Yes	203 (41.8%)	154 (38.9%)	49 (54.4%)	
VP:				0.192
No	155 (31.9%)	132 (33.3%)	23 (25.6%)	

Table S4: Coherent linear screening (VIF) in each queue

ICU-28Dead		Hosp_28Dead		external validation	
**Variable Names**	**VIF**	**Variable Names**	**VIF**	**Variable Names**	**VIF**
RAR	1.44	RAR	1.48	RAR	1.91
Age	1.89	Age	1.86	COPD	1.15
Gender	1.14	Gender	1.13	SOFA	2.68

Race	1.06	Race	1.06	APSIII	3.52
Weight	1.23	Weight	1.26	SAPSII	3.48
AKI	1.24	AKI	1.24	OASIS	2.21
DM	1.17	DM	1.15	APACHEII	3.97
HLD	1.11	HLD	1.09	TB	2.16
SOFA	3.21	SOFA	3.12	URE	1.53
APSIII	4.08	APSIII	4.12	CRRT	1.61
SIRS	1.26	SIRS	1.26	GC	1.06
SAPSII	4.32	SAPSII	4.37	RDW	2.14
OASIS	2.56	OASIS	2.61		
APACHEII	3.45	APACHEII	3.50		
HR	1.36	HR	1.35		
NBPS	1.14	NBPS	1.14		
RR	1.18	RR	1.19		
SPO2	1.08	SPO2	1.10		
temperaturef	1.04	temperaturef	1.04		
PLT	1.30	PLT	1.30		
RBC	1.38	RBC	1.35		
WBC	1.17	WBC	1.11		
Tca	1.24	Tca	1.20		
AG	2.25	AG	2.28		
Glu	1.20	Glu	1.23		
TCO2	1.63	TCO2	1.62		
Lac	2.38	Lac	2.38		
PH	1.50	PH	1.50		
PO2	1.15	PO2	1.14		

PT	1.31	PT	1.24
APTT	1.14	APTT	1.13
ALT	1.66	ALT	1.68
TB	1.51	TB	1.47
URE	1.93	URE	1.86
LDH	1.81	LDH	1.81
Mg	1.36	Mg	1.31
Lymphocyte count	1.16	Lymphocyte count	1.07
CRRT	1.40	CRRT	1.37
ventilation	1.08	ventilation	1.10
Sa	1.37	Sa	1.39
GC	1.09	GC	1.08
VP	1.36	VP	1.33

Table S5: ICU-28Dead multi factor Cox regression (modle3) proportional hazards assessment

**Variable Name**	**df**	**P-value**
Age	1	0.617
Gender	1	0.394
Race	1	0.070
Weight	1	0.879
AKI	1	0.479
DM	1	0.981

HLD	1	0.055
SOFA	1	0.013
APSIII	1	0.001
SIRS	1	0.283
SAPSII	1	0.001
OASIS	1	0.000
APACHEII	1	0.021
HR	1	0.250
NBPS	1	0.148
RR	1	0.672
SPO2	1	0.499
TF	1	0.177
PLT	1	0.185
RBC	1	0.258
WBC	1	0.991
AG	1	0.152
Tca	1	0.414
Glu	1	0.598
TCO2	1	0.675
Lac	1	0.004
PH	1	0.014
PO2	1	0.314
PT	1	0.232
PTT	1	0.670
ALT	1	0.026
TB	1	0.594

URE	1	0.564
LDH	1	0.000
Mg	1	0.549
Lym	1	0.383
CRRT	1	0.002
ventilation	1	0.423
Sa	1	0.005
GC	1	0.007
VP	1	0.419
RAR	1	0.558
GLOBAL	42	0.134

Note: AKI, Acute Kidney Injury; DM, Diabetes Mellitus; HLD, Hyperlipidemia; SOFA, Sequential Organ Failure Assessment; APS III, Acute Physiology Score III; SIRS, Systemic Inflammatory Response Syndrome; SAPS II, Simplified Acute Physiology Score II; OASIS, Oxford Acute Severity of Illness Score; APACHE II, Acute Physiology and Chronic Health Evaluation II; HR, Heart Rate; NBPS, Non-invasive Blood Pressure Systolic; RR, Respiratory Rate; SpO_2, Oxygen Saturation; TF, Temperature (Fahrenheit); PLT, Platelet Count; RBC, Red Blood Cell Count; WBC, White Blood Cell Count; AG, Anion Gap; Tca, Total Calcium; Glu, Glucose; TCO_2, Total Carbon Dioxide; Lac, Lactate; PH, Acidity and Alkalinity (pH); PO_2, Partial Pressure of Oxygen; PT, Prothrombin Time; PTT, Partial Thromboplastin Time; ALT, Alanine Aminotransferase; TB, Total Bilirubin; URE, Blood Urea Nitrogen; LDH, Lactate Dehydrogenase; Mg, Magnesium; Lym, Lymphocyte Count; CRRT, Continuous Renal Replacement Therapy; Ventilation, Mechanical Ventilation; Sa, Sedatives/Analgesics; GC, Glucocorticoids; VP, Vasopressors; RAR, Red Cell Distribution Width to Albumin Ratio

Table S6: Hosp-28Dead multi factor Cox regression (modle3) proportional hazards assessment

**Variable Name**	**df**	**P-value**
Age	1	0.029
Gender	1	0.936
Race	1	0.755
Weight	1	0.062
AKI	1	0.015
DM	1	0.734
HLD	1	0.002
SOFA	1	0.016
APSIII	1	0.083
SIRS	1	0.214
SAPSII	1	0.001
OASIS	1	0.001
APACHEII	1	0.001
HR	1	0.431
NBPS	1	0.767
RR	1	0.200
SPO2	1	0.208
TF	1	0.053
PLT	1	0.447
RBC	1	0.001
WBC	1	0.794
AG	1	0.001
Tca	1	0.038
Glu	1	0.994
TCO2	1	0.001

Lac	1	0.001
PH	1	0.001
PO2	1	0.386
PT	1	0.293
PTT	1	0.743
ALT	1	0.001
TB	1	0.325
URE	1	0.526
LDH	1	0.001
Mg	1	0.752
Lym	1	0.004
CRRT	1	0.631
ventilation	1	0.018
Sa	1	0.465
GC	1	0.003
VP	1	0.360
RAR	1	0.172
GLOBAL	42	0.089

Note: AKI, Acute Kidney Injury; DM, Diabetes Mellitus; HLD, Hyperlipidemia; SOFA, Sequential Organ Failure Assessment; APS III, Acute Physiology Score III; SIRS, Systemic Inflammatory Response Syndrome; SAPS II, Simplified Acute Physiology Score II; OASIS, Oxford Acute Severity of Illness Score; APACHE II, Acute Physiology and Chronic Health Evaluation II; HR, Heart Rate; NBPS, Non-invasive Blood Pressure Systolic; RR, Respiratory Rate; SpO_2, Oxygen Saturation; TF, Temperature (Fahrenheit); PLT, Platelet Count; RBC, Red Blood Cell Count; WBC, White Blood Cell Count; AG, Anion Gap; Tca, Total Calcium; Glu, Glucose; TCO_2, Total Carbon Dioxide; Lac, Lactate; PH, Acidity and Alkalinity (pH); PO_2, Partial Pressure of Oxygen; PT, Prothrombin Time; PTT, Partial Thromboplastin Time; ALT, Alanine Aminotransferase; TB, Total Bilirubin; URE, Blood Urea Nitrogen; LDH, Lactate Dehydrogenase; Mg, Magnesium; Lym, Lymphocyte Count; CRRT, Continuous Renal Replacement Therapy; Ventilation, Mechanical Ventilation; Sa, Sedatives/Analgesics; GC, Glucocorticoids; VP, Vasopressors; RAR, Red

Cell Distribution Width to Albumin Ratio
